# Global distribution patterns and niche modelling of the invasive *Kalanchoe* × *houghtonii* (Crassulaceae)

**DOI:** 10.1038/s41598-020-60079-2

**Published:** 2020-02-21

**Authors:** Sonia Herrando-Moraira, Daniel Vitales, Neus Nualart, Carlos Gómez-Bellver, Neus Ibáñez, Sergi Massó, Pilar Cachón-Ferrero, Pedro A. González-Gutiérrez, Daniel Guillot, Ileana Herrera, Daniel Shaw, Adriano Stinca, Zhiqiang Wang, Jordi López-Pujol

**Affiliations:** 1 Botanic Institute of Barcelona (IBB, CSIC-Ajuntament de Barcelona), 08038 Barcelona, Catalonia Spain; 2 Department of Evolutionary Biology, Ecology and Environmental Sciences, Faculty of Biology, University of Barcelona, 08028 Barcelona, Catalonia Spain; 3 Systematics and Evolution of Vascular Plants, Unit of Botany, Faculty of Biosciences, Autonomous University of Barcelona, 08193 Bellaterra, Catalonia Spain; 4 Centro de Investigaciones y Servicios Ambientales de Holguín, 80100 Holguín, Cuba; 5 Hortax, Cultivated Plant Taxonomy Group, 46118 Serra, Spain; 6 Universidad Espíritu Santo, Escuela de Ciencias Ambientales, 091650 Samborondón, Ecuador; 7 Department of Botany, National Institute of Biodiversity (INABIO), 170501 Quito, Ecuador; 8 School of Natural Sciences, Bangor University, LL57 2UW Bangor, Gwynedd United Kingdom; 9 Department of Environmental, Biological and Pharmaceutical Sciences and Technologies, University of Campania Luigi Vanvitelli, 81100 Caserta, Italy; 10 Institute for Advanced Study, Chengdu University, 610106 Chengdu, Sichuan China

**Keywords:** Invasive species, Invasive species

## Abstract

Invasive alien species are currently considered one of the main threats to global biodiversity. One of the most rapidly expanding invasive plants in recent times is *Kalanchoe* × *houghtonii* (Crassulaceae), an artificial hybrid created in the 1930s in the United States by experimental crossings between *K*. *daigremontiana* and *K*. *tubiflora*, two species endemic to Madagascar. Thanks to its large colonizing capacity (mainly derived from the production of asexual plantlets), *K*. × *houghtonii* soon escaped from cultivation and quickly spread in many parts of the world. However, its actual range is not well known due to the lack of a formal description until recent times (2006) and its strong morphological resemblance with one of its parentals (*K*. *daigremontiana*). The present study was aimed, in the first instance, to delimit the present distribution area of *K*. × *houghtonii* at the global scale by gathering and validating all its occurrences and to track its colonization history. Currently, *K*. × *houghtonii* can be found on all continents except Antarctica, although it did not reach a global distribution until the 2000s. Its potential distribution, estimated with MaxEnt modelling software, is mainly centered in subtropical regions, from 20° to 40° of both northern and southern latitudes, mostly in areas with a high anthropogenic activity. Unexpectedly, concomitant to a poleward migration, future niche models suggest a considerable reduction of its range by up to one-third compared to the present, which might be related with the Crassulaceaean Acid Metabolism (CAM) of *K*. × *houghtonii*. Further research may shed light as to whether a decrease in potential habitats constitutes a general pattern for Crassulaceae and CAM plants.

## Introduction

In recent decades, ornamental horticulture has become the major introduction pathway of naturalized and invasive alien plants^1–3^. A plant introduced for ornamental value, which has become a worldwide invader is *Kalanchoe* × *houghtonii* D. B. Ward [≡ *Bryophyllum* × *houghtonii* (D. B. Ward) P. I. Forst], an artificial hybrid obtained by the eminent horticulturist A. D. Houghton in the mid-1930s at his California greenhouses^4^, from the crossing of two species from the genus most frequent in cultivation, *Kalanchoe daigremontiana* Raym.-Hamet & H. Perrier and *Kalanchoe tubiflora* (Harv.) Raym.-Hamet. Both species are endemic to Madagascar but are today naturalized in many parts of the world with warm climates^5^. Despite this, it has been stated that this hybrid “arises spontaneously wherever these two species [*K*. *daigremontiana* and *K*. *tubiflora*] occur together”^6^ and Ward^7^ wrote “*Hybrida naturalis putativus*” in the protologue. However, we are still not aware of any explicit reports of spontaneous hybridization, despite the two parentals are often co-inhabiting. Indeed, we only know a few places where the three species can be found growing together (or separated by a few dozen to a few hundred meters): the historic downtown of Camagüey, Cuba (Pedro A. González-Gutiérrez, pers. observ.); the village of Catarroja in Spain^8^; the city of Bizerta, Tunisia^9^; and Lightning Ridge, Australia (see https://www.ala.org.au). Furthermore, according to the genetic data reported by Guerra-García *et al*.^10^, natural hybridization between *K*. *tubiflora* and *K*. *daigremontiana* in central Mexico would not have occurred despite a sympatric existence.

*Kalanchoe* × *houghtonii* is a perennial erect herb, generally monocarpic, that may reach up to 1.5  m (Supplementary Fig. S1). The leaves are opposite (or 3-verticillate), petiolate, with the leaf blade simple, from triangular to narrowly lanceolate, serrate, and mottled. It forms corymbiform inflorescences of often more than one hundred, pendulous, tetra- or penta-meric, dark-red flowers. It is distinguishable from the parental species by the leaf morphology and by the capacity of bulbil production (Supplementary Fig. S2). It has been reported that there were originally two clones of *K*. × *houghtonii* in cultivation, a fertile tetraploid and a sterile triploid^11–13^. It seems, however, that the original plants were triploid (2*n* = 51), as expected from the crossing between a diploid (*K*. *daigremontiana*, 2*n* = 34) and a tetraploid (*K*. *tubiflora*, 2*n* = 68) plant^14^.

Originally, the hybrid was named as *Bryophyllum tubimontanum* by Houghton^4^, however this name was not validly published. The taxon was also known as *Kalanchoe hybrida*^15^, an invalid name that has been widely used in horticulture (as *Kalanchoe* ‘Hybrida’) until the taxon was properly described in 2006^7^ (see Supplementary Text 1 for nomenclature details). The lack of proper nomenclature for the plant until very recently, in addition to its strong morphological resemblance with one of its parentals (*K*. *daigremontiana*), has allowed this plant to go unnoticed. Confusions between *K*. *daigremontiana* and *K*. × *houghtonii* have often been reported^16–18^ yet, even in modern studies the confusion continues such as in Venezuela where it is erroneously reported as *K*. *daigremontiana*^19,20^). Misidentifications may even go further as *K*. × *houghtonii* is sometimes confused with the other parental (*K*. *tubiflora*), a situation relatively common in some sources of information such as iNaturalist website (https://www.inaturalist.org). Even within the Chinese Academy of Sciences sponsored Plant Photo Bank of China (PPBC), many pictures that belong to *K*. × *houghtonii* have been placed under the morphologically distant *K*. *serrata* Mannoni & Boiteau. Therefore, the current distribution of *K*. × *houghtonii* is unknown and its invasive status could be much more serious than thought, with only two countries having declared this taxon as invasive (Australia and the United States). For instance, in Spain, where it is behaving as a very aggressive invader, this plant is neither included in the local floras nor listed in the national catalogue of alien invasive species. Only recently in Italy, this hybrid has been listed as naturalized in the national checklist of alien vascular flora^21^.

Despite its recent origin, *K*. × *houghtonii* is showing a tremendous invasive capability, probably resulting from some of the traits common to the genus, such as their drought-tolerance and easy propagation. New plants can be produced from almost any part of the mother plant, especially via means of clonal growth through the bulbils that arise from the leaf margins^6^ which, in suitable open locations (such as rocky or sandy places), quickly form dense stands, hence the popular name “mother of millions” or “mother of thousands”. Sexual reproduction seems to be rare in the nothospecies^7,19,20,22^, in accord with genetic data (one multilocus genotype has been found in all studied populations in central Mexico^10^). Where sexual reproduction exists such as in Cerro Saroche (Venezuela), it shows low success^19,20^. The successful spread of *K*. × *houghtonii* worldwide is, therefore, probably due to human transportation, which is closely linked to its growing use as a valued ornamental plant (^8,19^; J. López-Pujol *et al*., pers. obs.).

*Kalanchoe* × *houghtonii* can grow in a variety of habitats. These include all kind of urban and peri-urban environments; roofs, building façades, pavement cracks, roadsides, tree bases, waste grounds. They are also found in semi-natural and natural habitats such as sandy beaches, dry riverbeds, riparian areas, cliffs and rocky areas, shrublands, arid zones, forests and in some cases, it has even been identified as having an epiphytic habit (Supplementary Fig. S3). Our knowledge about the potential impact that *K*. × *houghtonii* could generate in these habitats is limited. Only at an arid zone in Venezuela, it has been reported that *K*. × *houghtonii* generates negative impact on cacti native richness^23^ and modify C and N pools in the soil^24^. This species could generate similar impacts in others habitats, which makes it a harmful invasive species. To suggest management options and predict vulnerable areas it is extremely important to detect which areas provide the most suitable habitats. Ecological niche modelling (ENM) represents one of most extended and useful quantitative methods to answer these issues^25,26^, especially considering its successful application to other invasive plant or animal studies^27^. An additional advantage of ENM is that models can be projected to several future climate change scenarios under the assumption of niche conservatism^28–31^. These spatial suitability models would be used as a basis to: (1) detect regions where sampling information gaps occur^32,33^; (2) allocate conservation efforts in currently occupied ranges^34–36^; or (3) identify areas at higher invasion risk through predictive modelling using present and projected future environmental conditions to allow preventive conservation measures^37–40^.

**Figure 1:**
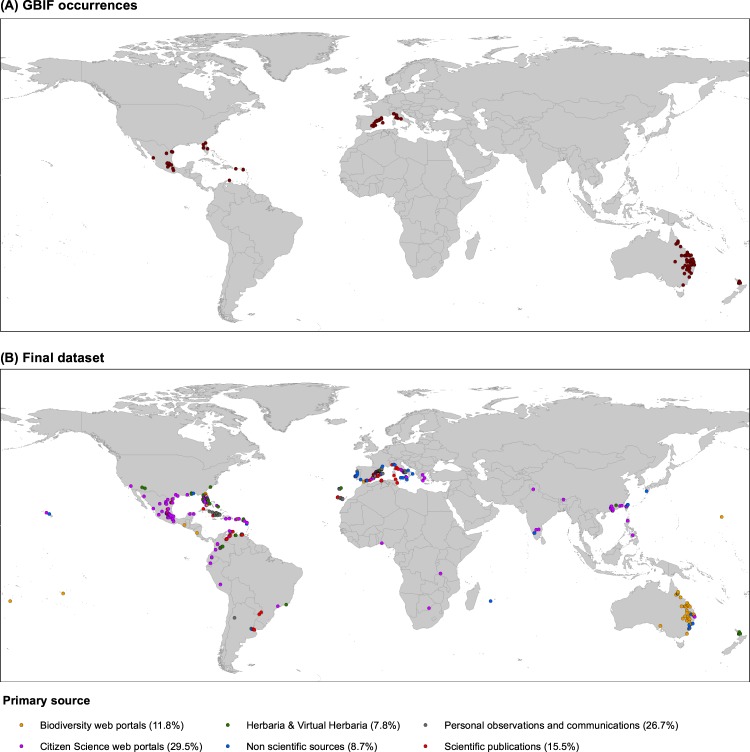
Geographic representation of occurrence records of *Kalanchoe* × *houghtonii* directly extracted from GBIF (**A**) and the Final dataset (**B**). Non-scientific resources include personal blogs, photography web portals, forums, newspapers and social media (see Supplementary Table S2).

For invasive species studies with ENM it is very important that an accurate occurrence database for the target species is created^26^. For the specific case of *K*. × *houghtonii*, there has been no comprehensive compilation of occurrence data which has in turn contributed to our lack of knowledge. This is likely due to several factors; its recent origin (mid-1930s) and description (2006), its taxonomic parental confusion, and its recent emergence as an ornamental plant at a global scale which is helping to its spread into many regions^8^. These factors have hindered the true extent of *K*. × *houghtonii*’s geographical distribution despite the many sources of gathered biodiversity spatial data that are currently available (see Fig. 1 in^41^). Key sources such as the occurrence data provided by citizen science databases, have been found to contain many spatial, environmental, temporal, and taxonomic biases^42,43^. The most common type of errors found in biodiversity data sources are: (1) duplicate records; (2) georeferenced occurrence points lacking precision (i.e. a low number of decimal places) or inaccurate occurrence points (i.e. datum was incorrectly specified); (3) geographic coordinates errors like switched latitude-longitude, sign confusions or mistakes amongst decimal/sexagesimal degrees; and (4) records outside continental ranges for terrestrial species; amongst others. Correcting these errors represents a critical point, since the creation of an accurate occurrence database for the target species has been reported to significantly change spatial inferences performed in biodiversity distribution studies (e.g.^44–46^).

The aims of this study were the following: (1) to explore the geography pattern of the distribution of *K*. × *houghtonii* globally, gathering all the occurrences, both published in standard publications and available from other sources; (2) to track its spatial and temporal colonizational history; (3) to predict the potential distribution of the nothospecies through ENM; (4) to test the contribution of the human activities to the ENM; (5) to test the impact of using different levels of occurrence data accuracy on ENM reconstructions; and (6) to predict how the distribution of this hybrid taxon is going to progress according to different scenarios of climate change.

## Methods

### Localities search and validation

The distribution range of *K*. × *houghtonii* was estimated by an extensive literature search that included: (1) major regional taxonomic works, checklists, lists and catalogues of naturalized and invasive plants; (2) research articles; (3) grey literature (e.g. technical reports); (4) major databases and information systems, including citizen science projects and digitized herbaria; (5) personal communications; and (6) personal blogs and other non-scientific websites (see Supplementary Text 2).

All citations were thoroughly validated one by one, keeping only those where an image was available showing the individuals in the wild or on herbaria sheets due the considerable confusion found between the hybrid and its parents, especially with *K*. *daigremontiana*. The only exceptions were citations published by taxonomic experts in the genus where correct identification could be ensured. Therefore, in addition to searching for citations under the name *K*. × *houghtonii* or any of its synonyms, it was necessary to check citations for the two parental species. Supplementary Table S1 includes all the names found in the original sources. It should be noted that 47.5% of occurrences were originally determined under other names or without proper indication of the species. In cases where geographic coordinates were not available, but a site description or a map location was provided, the first were inferred using Google Earth or a similar tool (e.g. Baidu Maps in the case of China). Localities with inaccurate descriptions or with geographic coordinates that offer very little precision (unable to discern with certainty the cell of 2.5 arc-min where the locality occurs, which is the selected spatial resolution of the variables used for ENM; see below) were not considered.

A total of 644 occurrences were gathered and validated in the first instance, which we regarded as the “Final” dataset. For all the occurrences, we collected the original determination of the plant, the country, the year of occurrence, and the primary source of the information (see Appendix). Where we found two or more occurrences for the same locality, we took only the information of the oldest occurrence to avoid duplication and because the oldest occurrence gave us the information of the first time the plant was recorded in a given site. Where no temporal occurrence data were available, we recorded the available publication date of the document or the website (and the “received date” for the specific cases of scientific papers).

To minimize the sampling bias effect for the ENM analysis, we followed the spatial filtering approach proposed by Kramer-Schadt *et al*.^47^, by retaining only one occurrence record within a buffer area of 20  km for the oversampled regions of *K*. × *houghtonii*: Mexico; Florida; eastern Iberian Peninsula; southern-central Italy; and Hong Kong. This spatially filtered dataset comprised 392 verified occurrences (hereafter “Final 20  km filtered” dataset).

### Environmental data and Ecological Niche Modelling (ENM)

In order to evaluate the potential distribution for *K*. × *houghtonii* we used the maximum entropy algorithm implemented in MaxEnt v.3.3.3k^48^, which only requires known species’ presence data and has proven to be a robust approach in comparison with other methods^49,50^. A set of 19 bioclimatic variables from WorldClim (http://www.worldclim.org/) v.1.4 at 2.5 arc-min (i.e. ca. 5  km) resolution covering the whole planet was used. Whilst finer resolutions are available (30 arc-sec), these may not be appropriate given uncertainties associated with geo-referencing approximate localities (a common situation for older herbarium records, especially for remote areas) or with geo-reference errors. In addition, we added the Human Footprint (HF), due to the fact that the establishment and spread of *K*. × *houghtonii* is often facilitated by human disturbance^10^. The variable HF is based on the anthropogenic impacts on the environment^51^, and created from nine global data layers covering human population pressure (population density), human land use, and infrastructure (built-up areas, night-time lights, and land use/land cover), and human access (coastlines, roads, railroads, and navigable rivers). A pairwise Pearson correlation analysis was conducted to retain six relative uncorrelated variables (*r* ≥ |0.85|), using the “SDM Toolbox” extension for ArcGIS^52^: maximum temperature of the warmest month (bio5); minimum temperature of the coldest month (bio6); mean temperature of the wettest quarter (bio8); precipitation of the wettest quarter (bio16); precipitation of the driest quarter (bio17); and precipitation of the coldest quarter (bio19). Variables were selected based on their relative contribution to the model (values of percent contribution, permutation importance, and jackknife regularized gaining train), and an exploration of curve response shape after running a “preliminary” model with MaxEnt. For this preliminary model we used all the occurrences of *K*. × *houghtonii*, all the variables (i.e. the 19 bioclimatic variables plus HF), 10 replicates, and 20% of the localities randomly selected to test the model.

The potential distribution for *K*. × *houghtonii* was estimated using two model sets, one for current conditions (1950–2000) and the second for future climatic conditions (year 2070). Climatic layers for the year 2070 were also downloaded from WorldClim database at the same resolution of 2.5 arc-min. In the first set of present conditions, we have distinguished between models that include the variable HF and those that do not (hereafter “with HF” and “without HF”, respectively) to test the contribution of human impacts on the potential distribution of this nothospecies. For the year 2070, we used three general circulation models that showed excellent performance among those that participated in the 5^th^ Coupled Model Inter-Comparison Project (CMIP5) experiment^53^: (1) the Community Climate System Model v.4 (CCSM4^54^); (2) the NOAA Geophysical Fluid Dynamics Laboratory Coupled Model 3 (GFDL-CM3^55^); and (3) the New Earth System Model of the Max Planck Institute for Meteorology (MPI-ESM-P: http://www.mpimet.mpg.de/en/science/models/mpi-esm/). In addition, projections of the three future models have been made with the two extreme scenarios of Representative Concentration Pathway (RCP), RCP 2.6 and RCP 8.5 (the first represents the most “benign” scenario, assuming a likely increase of 0.3–1.7 °C for ca. 2081–2100, whereas the latter or worst-case scenario predicts an increase of 2.6–4.8 °C^56^). Therefore, a total of six future models have been obtained corresponding to the different scenarios forecasted for 2070.

Definitive models were carried out in MaxEnt under “subsample” method (with 25% of localities randomly selected to test the model), using the final 20  km filtered dataset (392 records) as occurrence input, and with 100 replicates to ensure reliable results and a random sample of 10,000 background points. Model performance was evaluated using AUC (area under the curve of the receiver operating characteristic curve) values^57^ that ranges from 0.5 (no predictability) to 1 (perfect prediction)^58^, with values above 0.8 indicating a strong prediction^59^. In addition, we also calculated the true skill statistic (TSS) to validate the performance of the models. Values with a TSS higher than 0.5 are considered as optimal results in terms of power prediction^60^. To calculate the geographically suitable areas, the maximum sensitivity plus specificity (MSS) logistic threshold was used, a metric recommended for being very robust with all types of data^61^; MSS was used as the “cut-off” value to transform the continuous value outputs of MaxEnt to binary maps (absence/presence). The proportion of correctly predicted presence records (sensitivity) was calculated using this MSS threshold. Resulting models were visualized and processed using the software ArcGIS v.10.2.2 (ESRI, Redlands, California, USA). In order to track possible changes of the potential distribution of *K*. × *houghtonii* under climate change conditions, we geographically and climatically characterized the gained or lost areas in respect to future and present distributions. Such work was mainly carried out by intersecting binary maps (with the intersect ArcGIS tool), with the climatic values for each variable of the new layers obtained (overlap, lost, and gained) being extracted and plotted in a boxplot format with the ArcGIS graph tool.

### Occurrence data accuracy and its impacts upon potential distribution

To test the impact of using different levels of occurrence data accuracy in the performing ENMs, we prepared two different input datasets. The first, referred to as “Raw GBIF occurrence dataset”, was created by directly extracting species’ records deposited in Global Biodiversity Information Facility (GBIF; http://www.gbif.org/) under the names *Kalanchoe* × *houghtonii* and *Bryophyllum* × *houghtonii*, with consideration for all relevant data uploaded to the platform until June 2018. GBIF is the largest biodiversity data network in the world, containing hundreds of millions of species occurrence records, and it is the most used (and often the only) database to get occurrence records for ENM studies^62^. From the total records, only those with geographic coordinates were retained for further analysis (128 records). The second and “Final dataset” (644 records) had been previously checked by data quality, refined and compared to other data sources (see above how the data were extracted and validated). Both datasets were used as input records to perform the ENM analyses for present conditions using the same parameters specified above. For each occurrence dataset, two analyses were run; one using the six uncorrelated climatic variables and another adding the HF layer.

## Results

### Global occurrences of *Kalanchoe × houghtonii*: standard sources vs. citizen science

A close look at Fig. 1 and Supplementary Table S2 reveals that using exclusively “standard” sources (biodiversity web portals, herbaria, scientific publications, personal observations, and personal communications) allowed us to compile about 62% of the total occurrences (*N* = 644). Huge geographical regions and many countries are now included within the distributional range of *K*. × *houghtonii* due to “newer” sources, especially the citizen science web portals which provided 29.5% of the total occurrences (Fig. 1B; Supplementary Table S1). Truly non-scientific sources such as photo-upload tools (3.7%), social media platforms (0.2%), discussion forums (1.7%), general newspapers (0.3%), and even personal blogs (2.8%) have also provided valuable species occurrence data. Utilizing these newer sources, we found occurrences in several regions that would otherwise appear as “blank”: sub-Saharan Africa; the Indian Subcontinent; large parts of southern China; Japan; Philippines; large parts of both North (e.g. California, NW Mexico, Yucatán Peninsula, Dominican Republic) and South America (Peru and parts of Colombia, Ecuador, and Brazil); and also some parts of Europe (France and Greece).

### Temporal and geographic expansion of *Kalanchoe* × *houghtonii*

**Figure 2:**
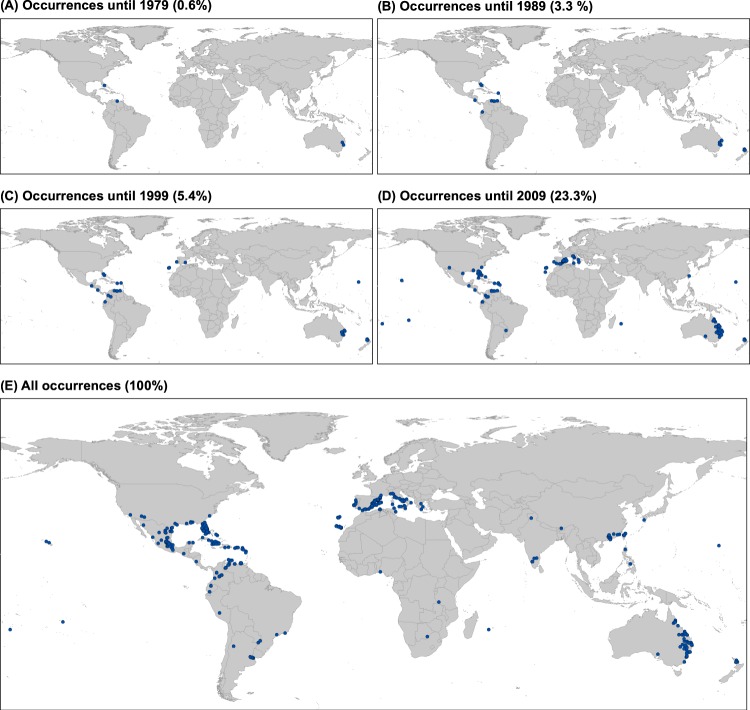
Geographic representation of the temporal evolution of occurrences recorded for *Kalanchoe* × *houghtonii* since 1979 to present with the indication of the accumulative percentage of occurrences for each decade.

Intercontinental colonization history of *K*. × *houghtonii* is shown in the Fig. 2. The first confirmed records of the hybrid were located as late as 1970 in Australia (that is, more than three decades after its obtainment). The only other records of *K*. × *houghtonii* from the 1970s were from the Bahamas and Venezuela (Fig. 2A). In the 1980s only a few new occurrences were registered, being located in nearby countries (New Zealand, Florida in the United States, Anguilla in Lesser Antilles, Nicaragua, and Ecuador; Fig. 2B). The species did not “jump” to other continents (Africa and Europe) until the 1990s, although the number of occurrences was still very small (until 1999, only 5.4% of the total records were registered; Fig. 2A–C). With the new millennium a great increase of the *K*. × *houghtonii* occurrences were observed, with all continents colonized except Antarctica (Fig. 2D). The largest increase in the distribution range of *K*. × *houghtonii* should be placed, however, during the most recent decade (2010–2019), providing up to 76.7% of the total records. The nothospecies is now reaching sub-Saharan Africa and the Indian Subcontinent, and thus now being present in most of the major tropical and subtropical latitudes of the world (Fig. 2E).

### Current potential distribution based upon climatic and anthropogenic pressures

The potential *K*. × *houghtonii* distribution model exhibited a strong predictive performance, as shown by the high AUC scores recovered (>0.95; Supplementary Table S3) and also TSS values (>0.79; Supplementary Table S3). This corresponds with the high percentage of occurrences correctly estimated inside suitable areas (sensitivity >90%; Supplementary Table S3).

The most explanatory variables to the models were in general equivalent for the different present occurrence datasets (Supplementary Table S3). When only climatic data were considered to perform the models, the variables with most influence in order of contribution according to the jackknife tests were bio6 (min. temp. of the coldest month), bio5 (max. temp. of the warmest month), and bio19 (precipitation of the coldest quarter). When HF data was used to perform the models, this variable showed the highest model contribution weight, even with scores above 50% regarding percent contribution (and above 20% regarding permutation importance; Supplementary Table S3). Current occurrences were, nevertheless, similarly well predicted inside suitable areas for both types of models, with HF (94.1%) or without HF (90.8%) (Supplementary Table S3).

**Figure 3:**
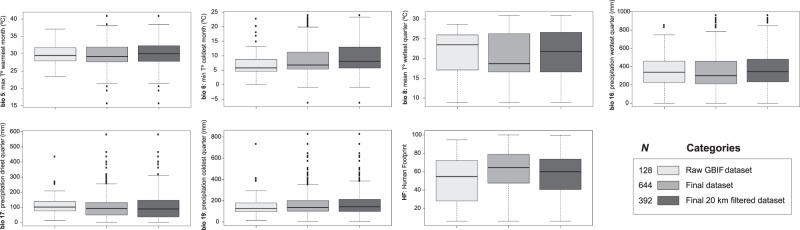
Boxplots representing values of the selected variables to construct the ENM models according to the three different categories of occurrence records datasets used. *N* = number of occurrences.

Our results indicated that *K*. × *houghtonii* presents a large temperature tolerance, although with a lower limit that generally does not fall below 0 °C during the coldest month and does not exceed 38 °C during the warmest month according to actual occurrence data (Fig. 3). The two most explanatory climatic variables were temperature extremes (bio5 and bio6; Supplementary Table S3), which highlight the sharp temperature tolerance limits of *K*. × *houghtonii*. Regarding precipitation, the nothospecies generally occurs in regions with low rainfall values, both in the driest and coldest quarters (bio17 and bio19, medians of approximately 100  mm and slightly higher than 100  mm, respectively; Fig. 3) but also in the wettest ones (bio16, medians around or slightly higher than 300  mm; Fig. 3).

**Figure 4:**
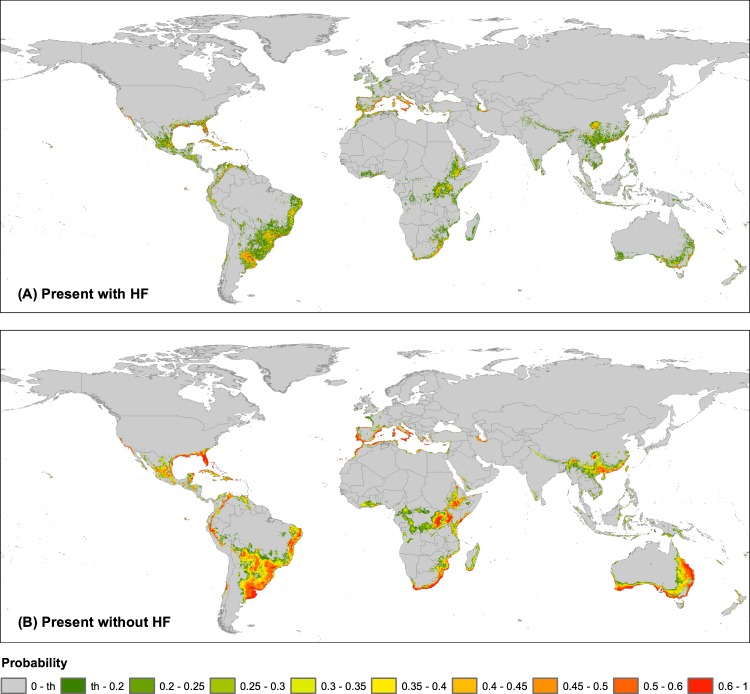
Potential distribution areas of *Kalanchoe* × *houghtonii* under present climatic conditions (**A**) including Human Footprint (HF) or (**B**) not including HF to perform the models. The input occurrence dataset used was the final 20  km filtered (392 records). In the legend, "th" means the selected threshold (see Methods for details).

**Figure 5:**
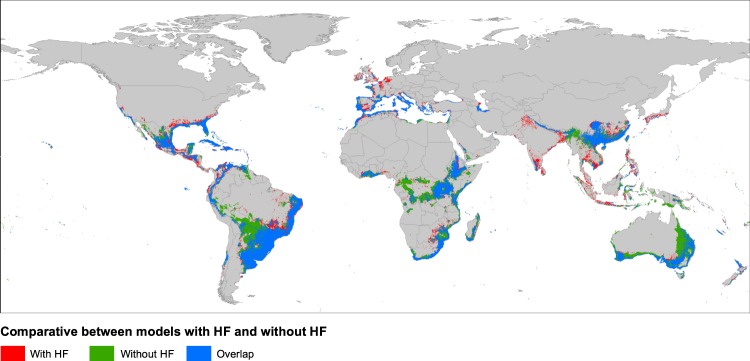
Comparison of potential distribution areas at present between models including Human Footprint (HF) variable (with HF) or not (without HF), showing overlapped ranges (blue), ranges only suitable with the model without HF (green), and ranges only suitable with the model with HF (red). The input occurrence dataset used was the final 20  km filtered (392 records).

**Figure 6:**
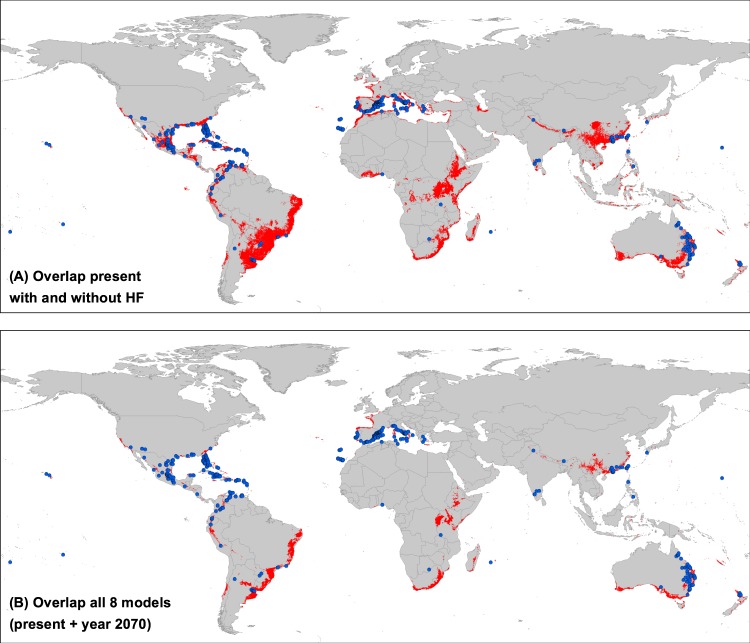
Representation of climatically potential suitable hotspot areas of *Kalanchoe* × *houghtonii*. (**A**) Overlapping regions of models performed with and without the Human Footprint (HF) variable. (**B**) Overlapping regions amongst all eight constructed models, showing the most suitable areas for the species at current and future time. Blue dots represent all the presence points currently known of the species, corresponding to the Final dataset (644 records).

The maps depicted in Fig. 4 showed how the occurrence probability is distributed globally. In general, suitable areas were located around Mediterranean and subtropical regions, from 20° to 40° of both northern and southern latitudes, although some tropical latitudes were also included. We detected that the model performed without HF predicted around 25% more suitable ranges than the model with HF (18.7 × 10^6^ km^2^ vs. 14.0 × 10^6^ km^2^; Table 1). These additional areas predicted as suitable can represent geographic regions with adequate climatic conditions for the nothospecies establishment (see red areas in Fig. 5), but with low human disturbances. Otherwise, we also detected some regions in the model with HF that can present appropriate human related requirements but are not recovered with the model exclusively using climate variables (see green areas in Fig. 5). Thus, it seems reasonable to consider that the most probable regions to be colonized are those with high suitability values using the two model types (with and without HF; see blue areas in Fig. 5), and could be regarded as possible invasion hotspots or “red alert” areas (see red areas in Fig. 6A).

**Table 1:** Extension of suitable predicted areas of *Kalanchoe* × *houghtonii* for each model. The overlap area between present (without considering Human Footprint variable; HF) and respective future models, and the lost and gained area for future predictions with respect to present models are also presented. Note that models were performed with the final occurrence dataset spatially filtered by 20  km (see text for details), which is composed by 392 presence points.

Model	Predicted area in km^2^	Difference with respect to present (model 1) in km^2^ (%)	Overlap with present (model 1) in km^2^ (%^a^; %^b^)	Lost area in the future with respect to model 1 in km^2^ (%)	Gained area in the future with respect to model 1 in km^2^ (%)
1. Present 20 km filtered (without HF)	18,682,228	—	—	—	—
2. Present 20 km filtered (with HF)	13,989,477	4,692,751 (−25.12)	11,425,388 (61.16; 81.67)	—	—
3. 2070 CCSM (RCP 2.6)	16,674,408	2,007,820 (−10.75)	14,504,213 (77.64; 86.98)	417,8015 (22.36)	2,170,195 (11.62)
4. 2070 CCSM (RCP 8.5)	11,467,657	7,214,571 (−38.62)	9,124,976 (48.84; 79.57)	9,557,252 (51.16)	2,342,681 (12.54)
5. 2070 GFDL (RCP 2.6)	12,555,141	6,127,087 (−32.80)	10,679,457 (57.16; 85.06)	8,002,771 (42.84)	1,875,684 (10.04)
6. 2070 GFDL (RCP 8.5)	9,611,927	9,070,301 (−48.55)	6,086,913 (32.58; 63.33)	12,595,315 (67.42)	3,525,014 (18.87)
7. 2070 MPI (RCP 2.6)	15,576,057	3,106,171 (−16.63)	13,222,720 (70.78; 84.89)	5,459,508 (29.22)	2,353,337 (12.60)
8. 2070 MPI (RCP 8.5)	8,713,360	9,968,868 (−53.36)	7,308,407 (39.12; 83.88)	11,373,821 (60.88)	1,404,953 (7.52)

^a^Percentage of overlapped area with respect to the area predicted with model 1.

^b^Percentage of overlapped area with respect to the area predicted with each assayed model (from 2 to 8).

These “red alert” areas should be considered, thus, new broad potential distribution regions over the five habitable continents where *K*. × *houghtonii* has not been cited so far (Fig. 6A). On the American continent, we found that suitable regions are mainly located around currently known species presence in North and Central America and the Caribbean. Whilst in South America large swathes of land, especially in the Atlantic Brazilian range, appear as potentially adequate zones where the nothospecies is currently absent. On the European continent, coastal areas of Mediterranean Basin showed high likelihood of occurrence, although in the northern Africa or eastern Mediterranean regions the nothospecies is not yet widely extended. Interestingly, some Atlantic coastlines in France and the UK exhibited suitable conditions, even though no specimens have been recorded to date. Eastern and Southern Africa proved to be one of the largest suitable regions detected yet contained a very low number of registered and confirmed presences. East Asia, especially Taiwan, south-central and south-eastern China, showed appropriate habitat suitability, even though the nothospecies presence is only verified in a narrow range. Notably, whilst there are no documented records in Malaysia, Indonesia, and Papua New Guinea, our models indicated suitable areas patchily distributed across the region. Finally, western, eastern and southern regions of Australia were all predicted as suitable despite the nothospecies almost exclusively being found in the eastern regions.

### Impact of occurrence data accuracy on potential distribution

**Figure 7:**
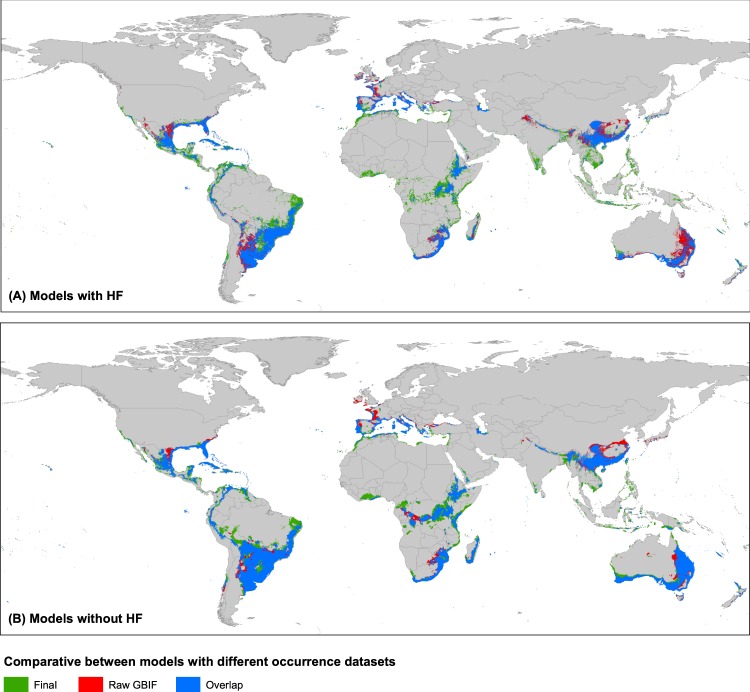
Comparison of potential distribution areas considering two input occurrence datasets: Final dataset (multi-source searching and verified; 644 records) and Raw GBIF dataset (directly downloaded from the platform; 128 records). Ecological niche models were performed at present for two input variable datasets (**A**) including Human Footprint (HF) or (**B**) without HF. The color ranges indicated: in blue, the overlapped areas between final and raw GBIF datasets; in green, suitable areas in models of final dataset; and in red, suitable areas in models of raw GBIF dataset.

The comparison of the extracted climatic and HF values for the different occurrence datasets used (Raw GBIF with 128 presences, Final with 644, and Final 20  km filtered with 392) did not reveal considerable differences, with extreme values being very similar (Fig. 3). The ENMs performed with Raw GBIF and Final occurrence datasets (Supplementary Figs. S4 and S5) highlighted large proportions of overlapping ranges (see blue areas in Fig. 7), representing intersections of 73.4–86.2% for models without HF and 63.3–73.6% for models with HF (data not shown). This may confirm the relatively low uncertainty and high robustness that the current models presented, since a 6-fold increase in the occurrence data did not greatly modify the global pattern of climatic suitability for *K*. × *houghtonii*. Additionally, 87.4% (model without HF) and 90.5% (model with HF) of final occurrences from a total of 644 (Table 2) were located inside suitable regions of Raw GBIF models; that is, even the poor and uncleaned occurrence dataset (128 records) was able to correctly predict most of the potential suitable areas where the species is confirmed to presently inhabit.

**Table 2:** Numeric comparisons of models performed with two combinations of input datasets: including the Human Footprint variable (with HF) or not (without HF); and using the occurrences of *Kalanchoe* × *houghtonii* of the Final dataset (multi-source searching and verified; 644 records) or the Raw GBIF dataset (directly download from the portal; 128 records).

Input variable dataset	Input occurrence dataset	Total predicted area (km^2^)	Overlap in predicted area Raw GBIF vs. Final (km^2^)	% of area that is not predicted by the other occurrence dataset model	% of final occurrences predicted in suitable area of Raw GBIF models
Without HF	Raw GBIF	15,339,714	13,229,145	13.8	87.4
	Final	18,028,866		26.6	—
With HF	Raw GBIF	11,725,301	8,631,877	26.4	90.5
	Final	13,636,498		36.7	—

However, the inspection of the suitable areas for *K*. × *houghtonii* recovered with the models employing the final dataset (both with and without HF) but that were not predicted using the Raw GBIF dataset (green ranges in Fig. 7; see also Table 2) gave somewhat unexpected results. Firstly, we observed suitable areas from equatorial climatic zones (according to the Köppen-Geiger classification; http://koeppen-geiger.vu-wien.ac.at/) in north-eastern South America, Africa, India, and SW Asia (Fig. 7). These areas, whilst not having any records in the Raw GBIF dataset, include some occurrences in the Final dataset (although these were very few). Accordingly, the Raw GBIF models may have ignored an outlier climate type where the species can also inhabit, in favor of an overrepresentation of subtropical and Mediterranean climates in warm temperate regions (http://koeppen-geiger.vu-wien.ac.at/). Secondly, tracking the source origin of the hybrid’s records located in these areas and in general for equatorial climatic zones, we realized that they were mostly gathered from citizen science portal websites like iNaturalist (see Fig. 1B), and not yet incorporated to GBIF.

The models performed with the Raw GBIF dataset also detected some regions not recovered with the Final dataset (red areas in Fig. 7; see Table 2). Indeed, these could be misleading areas, for which the Final occurrence models did not show positive signals of habitat suitability.

### Future potential distribution

**Figure 8:**
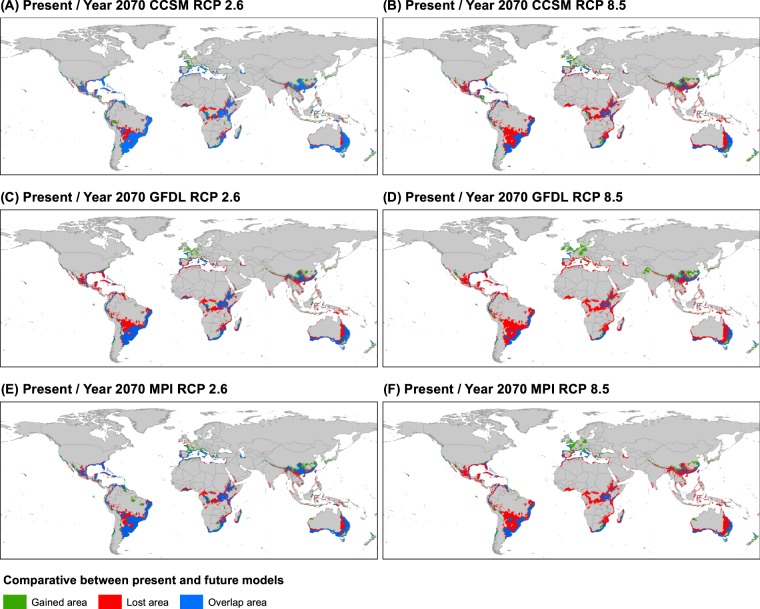
Comparison of potential distribution areas between present with future models, showing overlapped ranges (blue), gained ranges in future respect present model (green), and lost ranges in future respect present model (red). The input occurrence dataset used was the final 20  km filtered (392 records).

Under future climate scenarios (year 2070) our ENMs estimated a considerable reduction in the potential distribution area of the nothospecies with an average loss of 33.5% (±17.0%) or 6.3 × 10^6^ km2 (±3.2 × 10^6^ km2) with respect to present scenario data. The total predicted area decreased about 10.8–32.8% and 38.6–53.4% for RCP 2.6 (softest scenario) and RCP 8.5 (hardest scenario) models, respectively (Table 1; Supplementary Figs. S8–S10). When the current suitable areas were overlapped with those inferred in future projections, losses were even higher (22.4–42.8% in RCP 2.6 and 51.2–67.4% in RCP 8.5; Table 1). Consequently, a high number of known occurrences were located outside suitable areas under future conditions (11.2–30.9% in RCP 2.6 and 24.3–56.5% in RCP 8.5; Supplementary Table S3). In contrast, we identified some ranges that would be gained in the future (areas in green in Fig. 8; 10.0–12.6% in RCP 2.6 and 7.52–18.87% in RCP 8.5; Table 1).

## Discussion

### Global occurrences: standard sources vs. citizen science

Using exclusively “standard” sources including biodiversity web portals, herbaria, scientific publications, personal observations, and personal communications to search for documented locations would lead to large distributional biases. Indeed, using exclusively standard sources would imply that more than one third of them would be lost. Our results emphasize the role that citizen science plays in collecting biodiversity data, as generally agreed^63,64^. Citizen science web portals such as iNaturalist provided a large quantity of location data for *K*. *× houghonii* (Fig. 1B; Supplementary Table S1). Subsequently, such databases might constitute a primary source for biodiversity knowledge when high-quality photographs allowing for identification of the species and habitat or other voucher-essential data (collector, date, collection locality, and geographic coordinates) are provided to ensure the plant is wild and not cultivated.

### Colonization history: temporal and geographic expansion

It is significant that *K*. × *houghtonii* has reached a nearly worldwide distribution in just 80 years, being present on all the continents except Antarctica. Its creator, A. D. Houghton, already noted its potential as an experimental plant for genetic, commercial, and horticultural purposes^4^. Indeed, it seems that the plant was already common as a horticultural plant in the United States during late 1940s together with other species of the genus^14^. Experimental studies in *K*. × *houghtonii* outside of the United States were reported soon after, as those of F. Resende and colleagues carried out in Portugal in the 1950s aimed to study the consequences of hybridization^11,12^. From the 1980s, studies relating to their toxicity and medicinal potential started to be published (e.g.^65,66^). The first escapees of this plant into the wild are likely to have originated from research centers or horticultural greenhouses. In recent years, however, domestic gardens are the most apparent source of incursions into the wild, particularly due to its popularity as a garden plant, at least in North America^7^, Australia^67^, and Europe^8,16^. For example, in Spain and Italy, *K*. × *houghtonii* has become a serious and invasive problem, likely stemming from its popularity as an ornamental plant owing to its low maintenance (C. Gómez-Bellver and A. Stinca, pers. obs.). More recently it has become popular in China where it is commonly sold in markets (J. López-Pujol *et al*., pers. obs.).

Notwithstanding *K*. × *houghtonii*’s probable first escape from cultivation in the United States, the first recorded escapee(s) of this hybrid were from 1965 in Byrnestown (Queensland, Australia), albeit from an herbarium sheet without leaves (G. Brown, Queensland Herbarium, pers. comm.), so with uncertain identification as *K*. × *houghtonii*. The first record of the hybrid that we can confidently state as occurring in the wild is also from Australia, in this case from New South Wales collected in 1970 (Appendix). Although slightly earlier records may exist, as there are a few specimens dating from 1966–1969 also from Queensland, these were on loan at the time when we requested for scanned specimens. In Australia, *K*. × *houghtonii* soon became a serious invader and, by the late 2000s, it was already widespread throughout the eastern part of the country, reaching as far as southern regions (Fig. 2). At present it is listed as an invasive species in Australia^67–69^, where it often produces large infestations that can be accompanied by cattle poisoning episodes^65^. It has been present in New Zealand since at least the mid-1980s, but exclusively affecting the northern tip of the archipelago (Fig. 2B). The presence of the species is also confirmed in other parts of Oceania, including Micronesia (Wake Island) and Polynesia (Tonga, French Polynesia, and Hawaii; Fig. 2).

In addition to Oceania, America is the only region with occurrences confidently assigned to *K*. × *houghtonii* before the 1990s (Fig. 2B). The first recorded wild locality is from the Bahamas in 1975, as shown in a specimen preserved in the Steere Herbarium (NY 1515254; Appendix). Surprisingly, the first confident observation in the United States is as late as 1988 in Florida, although according to Mild^70^ the nothospecies would have been present in Harlingen (Texas) since the 1960s. Until the 2000s all occurrences were restricted to Florida, with its hypothetical expansion out of (but also within) this state having occurred in recent years. These increases appear to have coincided with its expansion in other parts of the world (Appendix; see also below). In Florida, this spread has merited its recent inclusion in the Florida Exotic Pest Plant Council Invasive Plant List^71^. In Venezuela, there are several records for the period 1979–1984 (Appendix). The presence of such ornamental plants escaping from cultivation in this country should not be regarded as exceptional, given the correlation between consumption of ornamental plants and gross domestic product (GDP) per capita^72^. Until the early 1980s, Venezuela was regarded as one of only four Latin American countries with an upper-middle-income economy^73^, with a GDP per capita higher than those of Portugal or South Korea (https://data.worldbank.org/). Although in some places of Central and South America (Anguilla, Colombia, Ecuador, Guatemala, Nicaragua, and Puerto Rico) the nothospecies was recorded before the year 2000, *K*. × *houghtonii* apparently had an “outbreak” in recent years (Appendix). Such an apparent explosion could have been due more to sociological reasons such as the rise in citizen science data collection and an increase in the use of ornamental plants in recent years throughout South America, rather than ecological or climatic ones. In Mexico, second only to the USA in the number of confirmed occurrences, all recorded localities of *K*. × *houghtonii* are much more recent (2012–2018), and are certainly linked to the launch of iNaturalist, with 66 out of 70 occurrences taken from this source (Appendix).

*Kalanchoe* × *houghtonii* did not apparently reach the Old World until more recently, with the first confirmed record in Europe being in 1996 from eastern Spain (Fig. 2C). Since then, the number of records in Europe and particularly on the Iberian Peninsula have increased exponentially (Appendix), partly because of the extensive network of local botanists but also due to its popularity in horticulture. However, this hybrid is not listed in the Spanish catalogue of invasive alien species. In Africa, the nothospecies would likely have arrived shortly after those to Spain, with the first observations being from Madeira Island in 1999, whilst the first occurrences for continental Africa coming almost a decade later, in Tunisia (Appendix). It should be noted the greater abundance in observations coming from the Portuguese (Madeira Archipelago) and Spanish (Canary Islands) outermost regions, in contrast to the scarcity of records for continental Africa. Such a disparity is likely due to the lower level of botanical knowledge on the African continent and particularly throughout sub-Saharan Africa^74,75^, combined with the low consumption of ornamental plants.

According to our data, Asia would be the last continent where *K*. × *houghtonii* spread to. All gathered Asian localities have been recorded since 2006 with a single exception; a population in Taipei, Taiwan Island (Fig. 2C; Appendix). The fact that many of the Chinese localities are from Hong Kong and Taiwan is not surprising, as these areas can be regarded as gateways for the introduction of alien species in China; being the primary places of first detection for many non-native species^76,77^.

Although a study aimed to discern whether the ecological niche of *K*. × *houghtonii* is broader than those of its parental species is underway, our field observations in Spain, where the three species are present, seems to suggest it; populations of *K*. × *houghtonii* are usually larger and more common than those of the parentals. Certainly, the nothospecies can produce large infestations, that in some cases may derive into dense monospecific phytocoenosis. Under favorable conditions (open, sunny areas under relatively dry or even semiarid conditions, on sandy or rocky soils; see below), densities may reach 1000–2000 individuals/m^2^ when plantlets and seedlings are produced^19,22^ (Supplementary Fig. S3E). These conditions probably mirror those of the parental species in Madagascar, where they grow on granite, sandstone or limestone outcrops, or coastal and inland unconsolidated sands^5,78,79^. In Cerro Saroche, Venezuela, a semiarid site with a typical xeric environment composed of spiny scrubs and thorny forests, the nothospecies has spread over an area of ca. 20  ha thanks to a very vigorous population growth (rate of growth, λ = 4.06), potentially allowing the population to quadruple in size every year. Such estimates are based on recruitment of asexual plantlets, due to the asexual plantlets ability to reproduce in less than one year. In contrast, sexual seedlings require a minimum of three years to reproduce^19^. According to Herrera *et al*.^19^, this strategy allows quick population growth during the initial phases of invasion, when populations are more susceptible to Allee effects or to post-introduction demographic bottlenecks. This strategy increases the probability of establishment and reduces the opportunities for effective control of *K*. × *houghtonii*. Similar results have been obtained for a population from Barranca de Metztitlán in Mexico, although for this population no sexually reproduced specimens were observed, and growth rates are lower than in Venezuela but still high (λ = 1.36)^22^. Thus, such a demographic strategy may have allowed the considerable global establishment of *K*. × *houghtonii*, as also occurs in other plants with a vegetative propagation strategy^80,81^.

### Potential distribution at present: climatic conditions and geographic regions where the species could inhabit

The most suitable conditions for *K*. × *houghtonii* are warm and dry climates. Low cold-tolerance is one of the most limiting factors of Crassulaceae and in general all succulent species with a CAM (Crassulacean Acid Metabolism) photosynthetic strategy. The lowest tolerance temperatures detected for this group of plants are −10 °C for *Opuntia ficus-indica* (L.) Mill. and −24 °C for *O*. *streptacantha* Lem.^82^. These two species, however, present special adaptations related to sugar accumulations that are necessary to prevent the intercellular ice crystal formation^82–84^, a set of specialized physiological traits that has not been observed in *K*. × *houghtonii*. The lack of sub-freezing acclimation during the coldest year periods (as bio6 showed; Fig. 3) could indeed be the most hindering factor for *K*. × *houghtonii* to reach higher latitudes, and greater altitudes.

*Kalanchoe* × *houghtonii* has a low water demand for its survival (Fig. 3). This is in accord with its ecophysiological traits, since CAM plants are highly tolerant and adapted to periodic droughts, salinity, or even elevated temperatures^85,86^. In fact, the stomatal opening during night-time instead of daytime offers a lower transpirational water loss^84^, which is a clear evolutionary adaptive advantage of increased water use efficiency and maintenance of considerable internal water reserves. However, it should be noted that the nothospecies would not be able to survive in geographic regions showing extremely irregular seasonal rains with extended periods of drought (e.g. semi-desert or desert). CAM plants need regular precipitation throughout the year or, at least, rains that are periodical and predictable; if they lose more than 50% of their total water reserves, they would perish^84,87–89^. Locations with too-high humidity (e.g. in tropical wet climates) would not be suitable for *K*. × *hougthonii* either, due to the stem and leaf cells being unable to release water fast enough into the atmosphere under such conditions. Once the maximum capacity of water storage is reached, plant cells may burst due to excesses of turgor pressure. Such an effect is well-described in succulent gardening when plants are over-watered, and their leaves may have a water-soaked, translucent appearance^90^. Nevertheless, precipitation of the coldest quarter (bio19) is the most important factor regarding the rainfall regime delimiting the potential distribution of *K*. × *hougthonii*. As this nothospecies does not have the sugar-mediated osmoregulation mechanism to control freeze dehydration of some low-temperature acclimated cacti^82^, a high-precipitation regime would produce the lowering of intracellular osmotic pressure, thus promoting the diffusion of intracellular water into the apoplastic spaces where ice crystals are formed^82^. The effects of cellular freeze dehydration is also a well-known phenomenon in succulents gardening—“weather that is most threatening to succulents is rain followed by frost”^91^. This is also the reason why watering should be restricted during winter in *Kalanchoe* spp. cultivation^6^.

### Antropogenic pressures impacting potential distribution

To our knowledge, *K*. × *hougthonii* shows the highest weight for HF in niche models (Supplementary Table S3) for any invasive plant; permutation importance in invasive plants is generally below 10%^92^. Such a high percentage is, however, not surprising given the species’ low dispersal capabilities; the overwhelming predominance of clonal growth through bulbils making the spread of *K*.× *hougthonii* a process strongly linked to humans. Undoubtedly, anthropogenic pressures influence the distribution of alien species in their non-native ranges along the several invasion stages (transport, introduction, establishment, and spread^93^), usually enhancing their expansion directly (e.g. deliberate or accidental releases^94^) or indirectly (e.g. via urbanization and land-use change^95^).

Initially, we expected the addition of the HF variable to ENMs would increase the suitable areas for *K*. × *houghtonii*, as previously detected for other cases such as *Acacia farnesiana* (L.) Willd^96^. In contrast, this expectation was not fulfilled in our case (the model performed without HF predicted around 25% more suitable ranges; Table 1 and Fig. 5). The presence of *K*. × *houghtonii* may be limited to the origin foci (mostly in private, and less-frequently, in public gardens) and adjoining areas, while climatically suitable regions far from human influence would not likely to be invaded. It should be noted that *K*. × *houghtonii* seems to be unable to disperse long distances, since sexual reproduction is quite unsuccessful: only a small proportion of produced seeds are viable (17.9%), with very low germination success (11.9%), and the survival rates of sexually-produced seedlings is also extremely low (10%^20^). In addition, the propagules mostly germinate *in situ*, just beneath or very near the mother plant. Indirect estimates of dispersal rates in Cerro Saroche (Venezuela) suggest that dispersal distances are generally limited^20^, although episodic floods that are produced during the rainy season can disperse the plantlets to relatively longer distances (I. Herrera, pers. obs.). Overall, the addition of HF variable to ENMs allowed us to obtain much more refined models for the potential distribution of the nothospecies (Fig. 5), which highlights the need to include this input element when distribution modelling for invasive species. The representation of *K*. × *houghtonii* occurrences on the map of “red alert” areas (Fig. 6A) suggests that the nothospecies could largely expand its current range.

### How do different levels of occurrence data accuracy influence ENM? Is it really necessary a multi-source search of occurrence data and an accurate filtering step to perform ENM?

In general, results shown in Table 2 suggest that a direct use of GBIF records could be appropriate without an extensive exploration of other sources and a taxonomic expertise validation process in covering the realized environmental species niche requirements. Nevertheless, it could be highly dependent on the target taxonomic group, as for primates, the comparison of datasets without or with expert knowledge resulted in the recovering of a higher number of outliers in non-expert group^97^.

Unexpectedly the ENMs performed with Raw GBIF and Final occurrence datasets resembled greatly (Table 2 and Fig. 7). In short, the resulting ENMs does not seem to be largely influenced by the occurrence data used, which might be due to the fact that species like *K*. × *houghtonii* and most Crassulaceae have a “very defined” interval range of climatic conditions; i.e. the tolerance to temperature and precipitation out of the optimal range is very low (see Fig. 3), and the shape of the response curves of bio17 and bio19 variables is more or less flat-topped and decline abruptly towards the margins (Supplementary Figs. S6 and S7). Conversely, in a study that tested the effect of spatial bias in GBIF records of Eurasian butterfly, the authors reported a decline of model quality with increased spatial bias^44^.

Careful observation of Fig. 7 shows, however, that there are areas not predicted using the Raw GBIF dataset (green ranges in Fig. 7), especially from equatorial climatic zones. This may be due to the fact that *K*. × *houghtonii* records located in these areas and in general for equatorial climatic zones are mostly gathered from citizen science websites (like iNaturalist; Fig. 1B) and not yet incorporated into GBIF. The lack of records from equatorial climatic zones on GBIF may simply respond to the nature of GBIF itself, as it is a research infrastructure funded by the world’s governments. Therefore, biodiversity data uploaded greatly depends on the node of every country. Many countries located in equatorial zones are non-participating countries in GBIF. This may be attributed to being low-income economies, although other reasons may apply (e.g. political). However, as noted by Amano and Sutherland^98^, even in participant countries, the amount of data provided may be low because the number of GBIF records per square kilometer depends greatly on GDP per capita (i.e. economic wealth), the number of English speakers, and country’s level of security. All these parameters rank generally very low in equatorial countries, which have hampered access to adequate funding for research centers and the work of researchers itself. Additionally, even in the case that citizen science portal websites are working well in a given country by providing lots of occurrences (e.g. PPBC in China), these data may not reach GBIF if there are no nodes submitting or endure long delays if nodes are not working properly.

Our results also show an overprediction of the nothospecies’ range with the Raw GBIF dataset (red areas in Fig. 7). Other studies comparing unfiltered vs. filtered presence datasets reported similar results. For example, false levels of species richness were recovered on large ecoregions for the plant tribe Cinchoneae (Rubiaceae)^99^ or wider elevational extents for three species of *Phaedranassa* (Amaryllidaceae)^100^.

A multi-source search and expert verification of presence records, aimed to avoid taxonomic (i.e. misidentifications) and spatial (i.e. georeferencing errors) biases, is generically regarded as critical to research focused on identification of current species location^101^ and evaluation of the conservation status of a given species (e.g.^102^). On the contrary, considering the results found here, one may think that these previous processes would not be necessary in ENM studies with a biogeographical global focus, as the “big picture” using Raw GBIF and final datasets is similar. Graham *et al*.^103^ also concluded that ENMs are particularly robust to moderate levels of errors in occurrence databases. Nevertheless, using exclusively GBIF data as a way to reduce time and money is only relatively justified for species distributed across well explored large-scale ranges and regions containing GBIF data providers. It should be noted that ENM approaches conducted for those species distributed in areas that do not provide data to GBIF, or that do not do it promptly, could be more susceptible to produce biased results. In addition, a detailed inspection of Fig. 7 shows that large regions of countries and even entire countries were not predicted as suitable using the Raw GBIF dataset, which eventually would have serious effects on the future management of *K*. × *houghtonii* in case of invasion; for example, regions or countries not suitable for the species would not implement policies of early detection/early warning. This suggests that making a complete multi-source location search, particularly including citizen science and other online databases, followed by a refining step is of critical importance in ENMs with management or conservation ends.

### Effects of climate change on global potential future distribution

A considerable reduction of *K*. × *houghtonii*’s current range (Table 1) could occur due to the lack of adequate climates, including many locations (Supplementary Table S3). The geographic regions predicted to be lost with respect to present scenarios is presented in Fig. 8 (red coloration). As a general pattern, it was observed that: (1) the lower the latitude, the more areas lost; and (2) most of the lost areas are continental, whereas many coastal areas are maintained. The latter observation agrees with multi-species studies of plant invaders in the United States (896 spp.)^104^ and Australia (72 spp.)^105^, and probably reflects that continental areas would tend to undergo more severe climatic changes than coastlines.

On the other hand, new suitable areas are predicted under future climate scenarios, which are mainly located in higher latitudes on both northern and southern hemispheres (Fig. 8). One clear example where the species cannot survive under present conditions is northern Europe, which would become climatically suitable. Such predicted latitudinal migration, with a poleward shift for *K*. × *houghtonii*, is interestingly following one of the most typical and well documented effects of climate change on species distribution^106,107^. High similarities were detected between results found here and other global assessments of future distributions of the top 100 worst invasive species performed by Bellard *et al*.^108^. According to these authors, tropical regions at low latitudes would experience the greatest decrease in potential number of invasive species, while the expanded ranges were in temperate regions such as northern Europe. In agreement with this, a recent study of 783 ornamental alien species planted in European gardens but not yet naturalized, observed that under a warming climate the hotspots of naturalization and invasion risk would considerably increase in northern and eastern parts of the European range^109^.

Considering the marked invasive behavior and the relatively wide temperature and precipitation ranges shown by the species (Fig. 3), one might expect future increase in its distribution range. On the contrary to this expectation, future projections suggest that the plant would be negatively affected by the ongoing climate change. Other global plant invaders have also shown potential future range contractions, for example a 32% reduction was observed for the grassland weed *Nassella neesiana* (Trin. & Rupr.) Barkworth due to increases in temperature leading to lethal heat stress^110^. As far as we know, the present study is the first estimate of global range shifts under a scenario of climate change not only for a Crassulaceae species but also for CAM species. Studies carried out at local or regional levels have yielded equivocal results: some show a range expansion such as *Kalanchoe* × *houghtonii* in NW Spain^16^ and *Echinocereus reichenbachii* (Terscheck ex Walp.) J. N. Haage in the United States^111^, whilst for others a range reduction was detected, e.g. *Kalanchoe tubiflora* and *Pereskia aculeata* Mill. in eastern Australia^112^ or *Coryphantha werdermannii* Boed. in northern Mexico^113^. Looking towards future research projects, it would be interesting to test whether a decrease in potential habitats might constitute a general pattern for Crassulaceae and CAM plants.

Shrinkage of potential areas for the year 2070 compared to the present in *K*. × *houghtonii* might be related to its ecophysiological traits. The extraction of climatic values of lost and gained areas in respect to future/present ranges revealed that *K*. × *houghtonii* would tend to be displaced to regions with low precipitation values. Inversely, regions with high rainfall values would be abandoned (see bio16, bio17, and bio19 in Supplementary Fig. S11). In contrast, the temperature ranges where it inhabits at present, both for minimum and maximum limits, would be maintained without significant modifications in the year 2070 (see bio5, bio6, and bio8 in Supplementary Fig. S11). As noted previously it seems that *K*. × *houghtonii* would not be able to withstand extended long wet periods, particularly during cold annual intervals. The combination of water storage saturation and freezing temperatures may injure succulent plants^82^. Thus, at least for *K*. × *houghtonii*, climatically suitable areas for the year 2070 are significantly reduced, because part of the current potential ranges would become unsuitable due to an excess of rainfall. This is in accordance with global trend changes, of which a significant rise in extreme periods of prolonged wet cycles have been estimated^114,115^.

Ultimately, we explored where the most likely potential invasion hotspots over present and future time slices could be located. This was achieved through the intersection of the eight ENMs performed which consisted of present scenario with and without HF plus six future scenarios (Fig. 6B). With this approach, firstly we observed that the main current presence locations of *K*. × *houghtonii* would still appear as suitable (e.g. Florida, Río de la Plata region, the Mediterranean Basin, the Chinese province of Guangdong, Taiwan Island, and western Australia). Secondly, we observed several areas where the species has not been documented to date but potentially have a high success rate of invasion. These areas could be regarded as “red alert areas”, and include California, central Chile coastline, most of the Atlantic coast of Brazil, Uruguay, northern Argentina, north-western Iberian Peninsula, the Atlantic coast of France, Corsica, Azores Islands, Crete, East African mountains, parts of Madagascar, South Africa, southern continental China, southern Australia, Tasmania, and New Caledonia (Fig. 6B).

### Conclusions and future prospects

In this study, we analyzed for the first time the global geographic distribution of the invasive plant *Kalanchoe* × *houghtonii*. The main conclusions drawn from this study are as follows: (1) a multi-source data compilation process was key in allowing us to infer the fast and complex expansion patterns shown by this hybrid; (2) the results of ENM (to our knowledge, the first one carried out with a CAM taxon on a worldwide scale) indicate that *K*. × *houghtonii* could expand largely on Mediterranean and subtropical regions of the planet; (3) the HF variable included in the modelling showed strikingly high contribution weights (even with scores above 50%), highlighting its close relationship with anthropic environments; and (4) a general reduction of the potential range of *K*. × *houghtonii* for the year 2070 can be anticipated. Considering the strong association with HF shown by *K*. × *houghtonii*, particular attention should be paid to the highly humanized regions to control potential invasion progress of this plant. Finally, this study paves the way to future research aiming to understand whether the hybridization process broaden the ecological niche of *K*. × *houghtonii* compared to its parental species.

## Supplementary information

Supplementary Material.

Appendix.
