# Supplementary material for: Global distribution patterns and niche modelling of the invasive Kalanchoe × houghtonii (Crassulaceae)
Source: Sci Rep. 2020 Feb 21;10:3143. doi: 10.1038/s41598-020-60079-2 (PMC7035272; doi:10.1038/s41598-020-60079-2)

**Supplementary Material:**

**Global distribution patterns and niche modelling of the invasive *Kalanchoe* × *houghtonii* (Crassulaceae)**

**Sonia Herrando-Moraira^1^, Daniel Vitales^1^, Neus Nualart^1^, Carlos Gómez-Bellver^2^, Neus Ibáñez^1^, Sergi Massó^3^, Pilar Cachón-Ferrero^1^, Pedro A. González-Gutiérrez^4^, Daniel Guillot^5^, Ileana Herrera^6,7^, Daniel Shaw^8^, Adriano Stinca^9^, Zhiqiang Wang^10^, Jordi López-Pujol^1^**

^1^Botanic Institute of Barcelona (IBB, CSIC-Ajuntament de Barcelona), 08038 Barcelona, Catalonia, Spain.

^2^Department of Evolutionary Biology, Ecology and Environmental Sciences, Faculty of Biology, University of Barcelona, 08028 Barcelona, Catalonia, Spain.

^3^Systematics and Evolution of Vascular Plants, Unit of Botany, Faculty of Biosciences, Autonomous University of Barcelona, 08193 Bellaterra, Catalonia, Spain.

^4^Centro de Investigaciones y Servicios Ambientales de Holguín, 80100 Holguín, Cuba.

^5^Hortax, Cultivated Plant Taxonomy Group, 46118 Serra, Spain.

^6^Universidad Espíritu Santo, Escuela de Ciencias Ambientales, 091650 Samborondón, Ecuador.

^7^Department of Botany, National Institute of Biodiversity (INABIO), 170501 Quito, Ecuador.

^8^School of Natural Sciences, Bangor University, LL57 2UW Bangor, Gwynedd, United Kingdom.

^9^Department of Environmental, Biological and Pharmaceutical Sciences and Technologies, University of Campania Luigi Vanvitelli, 81100 Caserta, Italy.

^10^Institute for Advanced Study, Chengdu University, 610106 Chengdu, Sichuan, China

Sonia Herrando-Moraira and Daniel Vitales contributed equally.

Correspondence and requests for materials should be addressed to Z.W. (email: wangzhiqiang@[cdu.edu.cn](http://cdu.edu.cn/)) or J.L.-P. (email: jlopez@ibb.csic.es)

**Supplementary Text 1**

**Nomenclatural considerations in the genus *Kalanchoe* and the studied species**

*Kalanchoe* Adanson, Fam. Pl. 2: 248. 1763

= *Bryophyllum* Salisb., Parad. Lond. pl. 3. 1805

= *Kitchingia* Baker, J. Linn. Soc., Bot. 18: 268. 1881

This genus is native mainly to Madagascar and east and south of Africa, extending to tropical Africa, Arabia and tropical and south-east of Asia. It was first named as *Kalanchoe* (“*Kalanchoè*”) by Michael Adanson in 1763 within the second volume of *Les Familles Naturelles des Plantes*, in 1763. In this book Adanson provides a table of morphological characters to discriminate the different genera within *Crassulaceae*, such as *Crassula*, C*otyledon* or *Kalanchoe* and also indicates that this genus come from China. In his herbarium he named as *Kalanchoè* a plant originally labeled as “*Cotyledon afra folia lato crasso laciniato flosculo aureo* Boerh. Ind.” (Chernetskyy 2011). This pre-Linnaean diagnostic phrase appears in *Species Plantarum* (Linnaeus 1753) as *Cotyledon laciniatus*, today the type specimen of the genus as *K. laciniata* (L.) DC.

The etymological origin may be due to a phonetic transcription from the Chinese “Kalan Chauhuy”, with the meaning “who falls and who grows”, probably referring to the bulbils (Boiteau & Allorge-Boiteau 1995). Or from ancient “kalanka-” (spot, rust) and “chaya” (gloss), perhaps referring to the glossy and sometimes reddish leaves of the Indian *K. laciniata* (Descoings 2003).

Two other names were proposed for naming the genus: *Bryophyllum* by Salisbury in 1806 and *Kitchingia* by Baker in 1881. The name *Kalanchoe* is now widely accepted to designate the genus and *Bryophyllum* and *Kitchingia* are regarded as synonyms. However, Berger (1930) recognized that the species included in *Kalanchoe* (*sensu lato*) actually belonged to two genera, whether to *Kalanchoe* (*sensu stricto*) or *Bryophyllum*. This latter idea was followed in the majority of recent floristic treatments (e.g., Fu et al. 2001; Moran 2009). The main argument was that species in *Bryophyllum* form propagules on the margin of the leaves while species of *Kalanchoe* do not form. It should be noted that the etymology of *Bryophyllum* is from the Greek “bryon/bryein” (sprout) and “phyllon” (leaf) and the type specimen is *B. pinnatum* (Lam.) Oken, synonym name of *K. pinnata* (Lam.) Pers).

The modern synoptic revision of *Kalanchoe* by Descoings (2003) provides two sections within the genus, despite the author admits that some species cannot be unambiguously placed in one of these two sections:

[1] Sect. *Kalanchoe*: Calyx with tube shorter than the lobes, often deeply divided to the base, sometimes the sepals almost free; lobes of the calyx generally appressed to the corolla tube; filaments appearing to be inserted at or above the middle of the calyx tube, rarely below; inflorescence with all or most of the flowers erect; leaves and inflorescences never with bulbils.

[2] Sect. *Bryophyllum* (Salisbury) Boiteau 1947 (incl. *Kitchingia* Baker): Calyx with tube longer than the lobes (sometimes ± of equal length), often ± inflated, generally not appressed to the corolla tube; filaments appearing to be inserted below the middle of the calyx tube, rarely above, rarely free or almost free; inflorescence with all or most of the flowers pendent; leaves and/or inflorescences of numerous taxa with bulbils.

More recently, the genus *Kalanchoe* has been recognized as consisting of three sections (Chernetskyy 2011) or three subgenres (Smith & Figueiredo 2018): *Bryophyllum*, *Kalanchoe* and *Kitchingia*.

We do not aware of spontaneous hybridization between sections. That would indicate a genetic barrier between two different groups, then presumably well defined. But we adopted the same conservative criterion of the majority of current authors (Boiteau & Allorge-Boiteau 1995; Descoings 2003; Chernetskyy 2011) of maintaining all species within *Kalanchoe*, mainly because in many cases the variability of the species and some intermediate morphological characters do not allow a clear assignment to one group or another. In this regard, it has been described an artificial hybridization between a species of *Kalanchoe* (seed parent) and a species of *Bryophyllum* (pollen parent) (Izumikawa et al. 2008), obtaining plants with intermediate characters and a lack of bulbil formation on the leaf margin.

*Kalanchoe daigremontiana* Raym.-Hamet & H. Perrier, Ann. Mus. Colon. Marseille, sér. 3, 2: 128–132. 1914

≡ *Bryophyllum daigremontianum* (Raym.-Hamet & H. Perrier) A. Berger. Nat. Pflanzenfam. (ed. 2) 18a: 412, fig. 197. 1930

Plant native to the southwest of Madagascar, the first specimens were collected in Mont Androhibolava, Marosavoha, Isalo and Makay (Boiteau & Allorge-Boiteau 1995). The epithet *daigremontiana* derives from Mr. and Mrs. Daigremont, prominent crassulacean collectors.

*Kalanchoe tubiflora* (Harv.) Raym.-Hamet, Beih. Bot. Centralbl. 29(2): 41. 1912

≡ *Bryophyllum tubiflorum* Harv., Fl. Cap. 2: 380. 1862

= *Kalanchoe delagoensis* Eckl. & Zeyh. Enum. Pl. Afr. Austral. 3: 305. 1836

Plant native to south of Madagascar, from Tulear to Fort Dauphin (Boiteau & Allorge-Boiteau 1995). The epithet *delagoense* derives from “Delagoabay”. The expedition leaded by W.F. Owen to survey the eastern coast of Africa was in Delagoa Bay in southern Mozambique in 1822. From there it sailed to Madagascar where the plant was probably collected and wrongly labelled as having originated from Mozambique (Figueiredo & Smith 2017).

Some botanists consider that the name *Kalanchoe delagoensis* have priority over the name *K. tubiflora* because *K. delagoensis* was considered as a *nomen nudum* by Hamet (1912). According this author, the Ecklon & Zeyher's descriptive statement associated with this name only gave “*Flores saturate rosei*” (dark pink flowers), doubtless a transcription of the information obtained from the collector of Owen's expedition, because it is impossible to have determined it from the dried specimen. Thus, this information could not satisfy the requirements for valid publication of a species name, and therefore the binomial proposed by Ecklon and Zeyher should be considered as a *nomen nudum*. Taking into account that the color of the flowers in *K. tubiflora* are somewhat variable and fairly similar than the other three species of *Kalanchoe* described in the publication of Ecklon & Zeyher, only the given character “*Flores saturate rosei*” appears to be a weak criterion to discriminate between the congeneric species. However, this nomenclatural problem is still open. Recently a request for a binding decision on the descriptive statement associated with *K. delagoensis* was made to General Committee of the International Association for Plant Taxonomy (Figueiredo & Smith 2017). Following different authors (Boiteau & Allorge-Boiteau 1995; Shaw 2008), in this paper we indicate this plant with the name *K. tubiflora*.

*Kalanchoe* × *houghtonii* D. B. Ward, Cact. Succ. J. 78(2): 94. 2006

≡ *Bryophyllum houghtonii* (D. B. Ward) P. I. Forst, Austrobaileya 7(2): 383. 2006

This is an artificial hybrid obtained by the eminent horticulturist A.D. Houghton in middle 1930s in his California greenhouses (Houghton 1935). This nothospecies comes from the crossing of two of the species of the genus most frequent in cultivation, *Kalanchoe daigremontiana* (seed parent) and *Kalanchoe tubiflora* (pollen parent).

Although Houghton named the hybrid *Bryophyllum tubimontanum*, this name was not validly published. The taxon was also named as *Kalanchoe hybrida* (Jacobsen 1954), an invalid name that has been widely used in horticulture (as *Kalanchoe* ‘Hybrida’ hort., *K*. ‘Houghton’s Hybrid’ or *K*. aff. ‘Hybrida’) until the taxon was properly described in 2006 as *K* × *houghtonii* (Ward 2006).

References

Chernetskyy M (2011) Problems in nomenclature and sysytematics in the subfamily *Kalanchoideae* (Crassulaceae) over the years. Acta Agrobot 64(4):67-74. https://doi.org/10.5586/aa.2011.047

Boiteau P (1947) Les plantes grasses de Madagascar. Cactus 12:5-10

Boiteau P, Allorge-Boiteau L (1995) *Kalanchoe* (Crassulacées) de Madagascar. Systématique, écophysiologie et phytochimie. Karthala, Paris

Descoings B (2003) *Kalanchoe*. In: Eggli U (ed) Illustrated handbook of succulent plants: Crassulaceae. Springer-Verlag, Berlin & Heidelberg, pp 143-181

Figueiredo E, Smith GF (2017) (56) Request for a binding decision on the descriptive statement associated with *Kalanchoe* *delagoensis* (Crassulaceae). Taxon 66(3):771

Fu K, Ohba H, Gilbert MG (2001) Crassulaceae. In: Flora of China. Science Press & Missouri Botanical Garden Press, Beijing & St. Louis

Hamet R (1912) Observations sur le *Kalanchoe* *tubiflora* nom. nov. Beihefte zum Botanischen Centralblatt. Original-Arbeiten. 2(29):41-44

Houghton AD (1935) An interesting hybrid. Cact Succ J 7:44

Izumikawa Y, Takei S, Nakamura I, Mii M (2008) Production and characterization of inter-sectional hybrids between *Kalanchoe spathulata* and *K*. *laxiflora* (= *Bryophyllum crenatum*). Euphytica 163:123-130. https://doi.org/10.1007/s10681-007-9619-8

Jacobsen, H (1954). Handbook of Succulent Plants 2. Blandford Press. London

Linnaeus C (1753) Species Plantarum. Impensis Laurentii Salvii, Stockholm

Moran RV (2009) Crassulaceae. In: Flora of North America. Oxford University Press, New York

Shaw JMH (2008) An investigation of the cultivated *Kalanchoe daigremontiana* group, with a checklist of Kalanchoe cultivars. Hanburyana 3:17-79

Smith, GF & Figueiredo, E (2018) The infrageneric classification and nomenclature of *Kalanchoe* Adans. (Crassulaceae), with special reference to the southern African species. Bradleya 36:162-172. https://doi.org/10.25223/brad.n36.2018.a10

Ward DB (2006) A name for a hybrid *Kalanchoe* now naturalized in Florida. Cact Succ J 78:92-95. https://doi.org/10.2985/0007-9367(2006)78[92:ANFAHK]2.0.CO;2

**Supplementary Text 2**

**Primary sources of *Kalanchoe* × *houghtonii* occurrences**

**Citizen science web portals with *Kalanchoe* × *houghtonii* occurrences**

- Biodiversidad Virtual (<https://www.biodiversidadvirtual.org/>)
- Botany pictures (<http://www.botanypictures.com>)
- Central african Plants, a Photo Guide. Biota Project (<http://www.centralafricanplants.senckenberg.de>)
- Chinese Field Herbarium (<http://www.cfh.ac.cn/>)
- efloraofindia (EFI) (<https://sites.google.com/site/efloraofindia/home>)
- iNaturalist.org ([https://www.inaturalist.org](https://www.inaturalist.org/))
- iSpot, share nature (<https://www.ispotnature.org>)
- India Biodiversity Portal (<https://indiabiodiversity.org/>)
- Plant Photo Bank of China (<http://ppbc.iplant.cn>)
- Project Noah (<http://www.projectnoah.org/spottings/6281785>)
- Tela Botanica (<https://www.tela-botanica.org>)

**Biodiversity web portals with *Kalanchoe* × *houghtonii* occurrences**

- Atlas of Living Australia (<https://www.ala.org.au/>)
- Australian Plant Image Index (<http://www.anbg.gov.au/photo/image-collection.html>)
- Early Detection and Distribution Mapping System (EDDMaps) (<https://www.eddmaps.org/florida/distribution/>)
- Flora Virtual Estación Biológica el Verde (<http://floraelverde.catec.upr.edu/index.php>)
- Flora of Gibraltar (<http://www.floraofgibraltar.myspecies.info/>[)](about:blank)
- Global Biodiversity Information Facility (GBIF) (<https://www.gbif.org/>)
- Moorea Biocode Databases. (<http://biocode.berkeley.edu>)
- Pacific Island Ecosystems at Risk (<http://www.hear.org/pier/>)
- Texas Invasives Database (<https://texasinvasives.org/invasives_database/>)
- The National Gardening Association database (<https://garden.org/plants/group/>)
- Tropicos (<http://www.tropicos.org>)

**Virtual herbaria with *Kalanchoe* × *houghtonii* occurrences**

- Alabama Plant Atlas (<http://www.floraofalabama.org/>)
- Atlas of Florida Plants (<http://www.florida.plantatlas.usf.edu>)
- Auckland Museum (<http://www.aucklandmuseum.com/collections-research/collections>)
- Chinese Virtual Herbarium (CVH) (<http://www.cvh.ac.cn>)
- Colecciones Científicas en Línea del Instituto de Ciencias Naturales (ICN) de la Universidad Nacional de Colombia (<http://www.biovirtual.unal.edu.co>)
- Florida Museum of Natural History (<https://www.floridamuseum.ufl.edu/collections/databases/>)
- Intermountain Region Herbarium Network (<http://intermountainbiota.org/portal/collections/>)
- New York Botanic Garden (<http://sweetgum.nybg.org>)
- Reflora - Brazilian Plants (<http://reflora.jbrj.gov.br>)
- Smithsonian Institution. National Museum of Natural History (<https://collections.nmnh.si.edu/search/>)

**Scientific publications (papers or books) with *Kalanchoe* × *houghtonii* occurrences**

Aymerich P (2015) Nuevos datos sobre plantas suculentas alóctonas en Cataluña. Bouteloua 22:99–116

Aymerich P (2016) Notas sobre plantas alóctonas de origen ornamental en el litoral septentrional de Cataluña. Bouteloua 26:78–91

Aymerich P, Gustamante L (2015) Nuevas citas de plantas alóctonas de origen ornamental en el litoral meridional de Cataluña. Bouteloua 20:22–41

Aymerich P, Gustamante L (2016) Nuevas citas de plantas alóctonas de origen ornamental en el litoral meridional de Cataluña, II. Bouteloua 24:93–112

Galasso G (2014) 217. *Kalanchoë* ×*houghtonii* D.B. Ward. In: Nepi C, Peccenini S, Peruzzi L (eds) Notulae alla checklist della Flora vascolare Italiana: 17 (2027 - 2070). Inform Bot Ital 46(1):71–86

García-González A, Riverón-Giró FB, Gómez JL, Hernández Y, Escalona R (2014) Plantas suculentas presentes en la comunidad de Cabo Cruz, Parque Nacional Desembarco del Granma, Cuba. Rev Cubana Cien Biol 4(2):94–100

García-Rivas A, Mori GM, González-Carcacía J, Rojas-Sandoval J, Pereira de Souza A, Mavárez J, Nassar JM (2012) Ensayos preliminares con microsatélites de *Kalanchoe daigremontiana* sugieren baja variabilidad genética en localidades colonizadas de la Región Caribeña. Bol Red Latinoam Estud Plant Invasoras 2(2):4–11

Giménez M (2012) Estudi de l’efecte de la flora invasora sobre les espècies autòctones del litoral de Llançà. Ann Inst Estud Empordanesos 43:301–325

Greuter W, Raus T (eds) (2012) Med-Checklist Notulae, 31. Willdenowia 42(2):287–295

Guerra-García A, Golubov J, Mandujano MC (2015) Invasion of *Kalanchoe* by clonal spread. *Biol Invasions* 17(6):1615–1622

Guillot D (2003) Acerca de cuatro taxones del género *Kalanchoe* Adanson en la Comunidad Valenciana (España). Blancoana 20:57–59

Guillot D (2008) Un nuevo taxón invasor para la flora balear, *Kalanchoe* × *houghtonii* D. B. Ward. Acta Bot Barc 51:129–130

Guillot D, Laguna E, López-Pujol J, Sáez Ll, Puche C (2014) *Kalanchoe* × *houghtonii* ‘Garbí’. Bouteloua 19:99–128

Guillot D, Laguna E, Rossello JA (2009) La familia Crassulaceae en la flora alóctona valenciana (Monografías de la revista Bouteloua, 4). Jolube Consultor y Editor Ambiental, Teruel y Jaca

Guillot D, Rosselló JA (2005) *Kalanchoe* x *hybrida* Jacobs., un nuevo taxón invasor en la Comunidad Valenciana. Lagascalia 25:176–177

Guillot D, Sáez L (2014) Algunas citas de neófitos de la isla de Mallorca. Bouteloua 17:135–144

Jiménez BA, Corona C, Campos K, Zaragoza G (2013) Retiro de plantas exóticas (*Kalanchoe delagoensis*) en la A13. In: Secretaría Ejecutiva de la Reserva Ecológica del Pedregal de San Ángel. Programa de Servicio Social. UNAM. Ciudad Universitaria, México D.F.

Herrera I, Ferrer-Paris JR, Hernández-Rosas JI, Nassar JM (2016) Impact of two invasive succulents on native-seedling recruitment in Neotropical arid environments. J Arid Environ 132:15–25

Hurrell JA, Delucchi G, Keller HA, Stampella PC, Guerrero EL (2012) *Bryophyllum* (Crassulaceae): especies ornamentales naturalizadas en la Argentina. Bonplandia 21(2):169–181

Laguna E, Guillot D, Rosselló R, Gómez MA, Ferrer PP, Deltoro V, Pérez P (2014) Nuevas citas de plantas alóctonas suculentas asilvestradas en la Comunidad Valenciana. Bouteloua 18:141–159

Lazzaro L, Ferretti G, Galasso G, Lastrucci L, Foggi B (2013) Contributo alla conoscenza della flora esotica dell Arcipelago Toscano, Italia. Atti Soc It Sci Nat Mus Civ Stor Nat Milano 154(1):3–24

Mild C (2013) Invasive exotics in the LRGV. Sabal 30(8):2–6

Otto R, Veerlove F (2016) New xenophytes from La Palma (Canary Islands, Spain), with emphasis on naturalized and (potentially) invasive species. Collect Bot 35:e001

Patiño G, Natacha C (2012) Inventario Florístico en Arbustales Xerófilos en la Localidad de Guayacán, Vertiente Norte de la Península de Araya, Estado Sucre, Venezuela. PhD Thesis. Universidad de Oriente

Palerm JC, Benoit C, Torres C, Vericad M, Valverde N (2011) *Kalanchoe daigremontiana* Hamet & Perrier (o *Kalanchoe* × *houghtonii* D. B. Ward.), un nou invasor agressiu al Parc natural de ses Salines d’Eivissa i Formentera a l’illa d’Eivissa. Boll Soc Hist Nat Balears 54:85–93

Pérez-García FJ, Jiménez-Sánchez ML, Garrido-Becerra JA, Martínez-Hernández F, Medina-Cazorla JM, Mendoza-Fernández A, Navarro-Pastor J, Rodríguez-Tamayo ML, Sola AJ, Mota JF (2008) Aportaciones al catálogo xenofítico de la provincia de Almería (Sureste Ibérico, España). Anales Biol 30:9–15

Pyke S (2008) Contribución al conocimiento de la flora alóctona catalana. Collect Bot 27:95–104

Podda L, Lazzeri V, Mayoral O, Bacchetta G (2012). The Checklist of the Sardinian Alien Flora: an Update. Not Bot Horti Agrobot Cluj Napoca 40(2):14–21

Roselló R (2009) Flora rupícola borrianenca (i II). Buris-ana 206:25–28

Saralegui Boza H, Álvarez Sorí D, Cuza Pérez A (2008) Las plantas y el deterioro de edificaciones no patrimoniales del Centro Histórico de la Habana Vieja. Revista Jard Bot Nac Univ Habana 29: 145–150

Senar R (2016) Nuevos datos para la flora alóctona valenciana. Bouteloua 23:118–140

Smith GF, Figueiredo E, Silva V (2015). *Kalanchoe × houghtonii* (Crassulaceae) recorded near Lisbon, Portugal. Bouteloua 20:97–99

Smith GF, Figueiredo E, Silva V (2015) Alien succulents naturalised and cultivated on the central west coast of Portugal. Bradleya 33:58–82

Stinca A, D’Auria G, Salerno G, Motti R (2013). Ulteriori integrazioni alla flora vascolare aliena della Campania (Sud Italia). Inform Bot Ital 45(1):71–81

Stinca A, Motti R (2009) The vascular flora of the Royal Park of Portici (Naples, Italy). Webbia 64(2):235–266

Stinca A, Motti R (2013) Aggiornamenti floristici per il Somma-Vesuvio e l’Isola di Capri (Campania, Sud Italia). Inform Bot Ital 45(1):35–43

Sukhorukov AP, Kushunina M, El Mokni R, Sáez Ll, El Aouni MH, Thomas FD (2018) Chorological and taxonomic notes on African plants, 3. Bot Lett 165(2):228–240

Sykes WR (1992) Succulent plants on Rangitoto Island. Auckland Bot Soc J 47(1):6–16

Torres L, Royo F, Arasa A (2003) Plantes vasculars del quadrat UTM 31TBF81, Santa Bàrbara (ORCA: Catàlegs Florístics Locals, 15). Institut d’Estudis Catalans, Barcelona

Van der Burg WJ, de Freitas J, Debrot AO, Lotz LAP (2012) Naturalised and invasive alien plant species in the Caribbean Netherlands: status, distribution, threats, priorities and recommendations. Report of a joint Imares/Carmabi/PRI project financed by the Dutch
Ministry of Economic Affairs, Agriculture & Innovation. Plant Research International, part of Wageningen UR PRI report 437.

Zimmer E (2015) A review of the succulent flora of Rangitoto Island, Hauraki Gulf, New Zealand. Xerophilia 5:3–139

**Personal blogs with *Kalanchoe houghtonii* occurrences**

- Biodiversidad Costa Granadina (2017) Kalanchoe daigremontiana x Kalanchoe delagoensis. In: Biodiversidad Costa Granadina y… (Plantas y Hongos)

<https://biocostagranadina.blogspot.com/2017/02/al-borde-de-un-sendero-en-caminos-de-la.html?showComment=1490040105930#c52731262839979720>

- Cifuentes F et al. (2015) A natural xerigarden. In: Aulets

<http://www.aulets.net/aulets-news/2015/2/24/a-natural-xerigarden>

- Clamote F (2015) Bryophyllum daigremontianum In: Plantas: Beleza e Diversidade <http://obotanicoaprendiznaterradosespantos.blogspot.com/2015/03/bryophyllum-daigremontianum.html>
- Faroleco (2012) Kalanchoe delagoensis. In: Faroleco As Plantas do Farol num ambiente ecológico... Farol + Eco só podia originar Faroleco

<http://faroleco.blogspot.com/2012/11/kolanchoe-delagoensis.html>

- Gauss J (2011) Flora of Ecuador’s Sierra. In: Ecuador Life and Culture

<https://ecuadorlifeandculture.wordpress.com/2011/11/22/flora-of-ecuadors-sierra/>

- Geni (2009) Mãe de milhares. In: Ciranda do Desenho

<http://cirandadodesenho.blogspot.com/2010/01/mae-de-milhares.html>

- Guerrero AM (2014) Flora de Quito (i - Li): Flora de la parroquia Pomasqui. In: Ecuador: personajes y especies (Galápagos y Continente)

<http://pinzonesygorriones.blogspot.com/2014/05/flora-de-quito-i-li-flora-de-la.html>

- Ice Rain-florist (2013) Prince Mountain Flowers. In: Ice Rain florist blog

<http://blog.sina.com.cn/s/blog_5cac4ff40102ercu.html>

- Marchetti C, Sacconi L (2011). Kalanchoe daigremontiana. In: Fiori e foglie… una pianta al giorno

<http://lnx.macalu.it/blog-fioriefoglie/2011/09/kalanchoe-daigremontiana/>

- Matchett W (2011) McLarty Museum. In: Space coast wildflowers. A growing collection of wildflower reference photos for Brevard county, Florida

<http://www.spacecoastwildflowers.com/2011/03/mclarty-museum-march-4-2011.html>

- Mt Gravatt Environment Group (2014) Our Bushland is in Our Hands

<https://megoutlook.org/2014/12/23/our-bushland-is-in-our-hands/>

- Mushimura (2013) Succulent plant. In: Diary of Mushimura

<http://d.hatena.ne.jp/kazuyo1014/touch/searchdiary?word=*%5B%A1%A1%C2%BF%C6%F9%BF%A2%CA%AA%5D>

- Sokolovsky S (2014) Plantas de Lujan (Buenos Aires)

<http://plantaslujan-k.blogspot.com/>

- Anonymous (undated) Terre de Haut. Le Chameau. In: Balades et écosystèmes de Guadeloupe

<http://www.guadeloupensites.com/le-chameau.php#1123>

- Sedore D, DeLeon S (undated) Devil’s Backbone. In: Wild South Florida <http://www.wildsouthflorida.com/devils.backbone.html>)
- Wagman J (2017) Devil’s Backbone. In: Plants of the Rio Grande Valley

<http://johnwagman.com/rgvnp/Devils%20Backbone%20-%20Kalanchoe%20daigremontiana/>

- ZG (2014) Kalanchoe daigremontiana Raym.-Hamet & Perret (Crassulaceae). In: Das plantas e das pessoas Um blogue de e para naturalistas que amam as plantas

<http://plantas-e-pessoas.blogspot.com/>

- Buddhist allegorical all around everywhere (2011) *Kalanchoe*. In: Buddhist allegorical all around

<https://blog.xuite.net/a20130909/twblog1/137423000-%E4%B8%8D%E6%AD%BB%E9%B3%A5%E6%B4%8B%E5%90%8A%E9%90%98>

**Photography web portals with *Kalanchoe houghtonii* occurrences**

- Bisque (<https://bisque.cyverse.org>)
- Dreamstime (<https://www.dreamstime.com/>)
- Flickr ([https://www.flickr.com](https://www.flickr.com/))
- Fotostock (<https://www.agefotostock.com/age/en/>)

**Online forums with *Kalanchoe houghtonii* occurrences**

- Forum Acta Plantarum (<http://www.actaplantarum.org/floraitaliae/>)
- FJpower (<http://fjpower.forumgratuit.org/>)
- Natural Photography Center ([http://nc.biodiv.tw/](http://nc.biodiv.tw/bbs/showthread.php?t=45086%E4%B8%8D%E6%AD%BB%E9%B3%A5))

**Newspapers with *Kalanchoe houghtonii* occurrences**

- Huelva información. Arrancan 720 kilos de una planta invasora en la playa del Espigón. 18/03/2008

[https://www.huelvainformacion.es/huelva/Arrancan-kilos-planta-invasora-Espigon_0_132586921.html#](https://www.huelvainformacion.es/huelva/Arrancan-kilos-planta-invasora-Espigon_0_132586921.html)!

- Diario de Ibiza. La mala hierba. 08/04/2016.

<https://www.diariodeibiza.es/pitiuses-balears/2016/04/08/mala-hierba/834195.html>

**Non online herbarium with *Kalanchoe houghtonii* occurrences**

- BC herbarium. Herbari de l’Institut Botànic de Barcelona
- BRI herbarium. Queensland Herbarium
- VEN herbarium. Herbario Nacional de Venezuela
- MADJ herbarium. Herbário do Jardim Botânico da Madeira
- QCA herbarium. Herbario QCA de la Pontificia Universidad Católica del Ecuador
- PAL herbarium. Erbario dell'Orto Botanico dell'Università degli Studi di Palermo

**Supplementary Tables and Figures**

**Table S1.** Number of occurrence records found for *Kalanchoe* × *houghtonii* under different names (synonyms or taxonomic confusions).

| **Name in the original source** | **Occurrences (%)** |
| --- | --- |
| *K*. × *houghtonii*, *B*. × *houghtonii*, *K*. × *hybrida* or parentals indication | 338 (52.5) |
| *K*. *daigremontiana* or *B*. *daigremontianum* | 155 (24.1) |
| *K*. *delagoense* or *B*. *delagoense* | 61 (9.5) |
| *K*. *tubiflora* or *B*. *tubiflorum* | 16 (2.5) |
| *K*. *serrata* or *B*. *serratum* | 8 (1.2) |
| *B*. *pinnatum* | 1 (0.2) |
| *K*. *rosei* | 1 (0.2) |
| *Kalanchoe* sp. or *Bryophyllum* sp. | 54 (8.4) |
| Mother of millions | 2 (0.3) |
| Chandelier plant | 1 (0.2) |
| ND | 6 (0.9) |

**Table S2.** Number of occurrence records of *Kalanchoe* × *houghtonii* extracted from the different data sources searched.

| **Sources** | **Occurrences (%)** |
| --- | --- |
| CITIZEN SCIENCE WEB PORTALS   - iNaturalist - Project Noah - Biodiversidad Virtual - Plant Photo Bank of China (PPBC) - Chinese Field Herbarium (CFH) - Botanypictures.com - Tela Botanica - efloraofindia (eFI) - iSpot - Central African Plants. A photo Guide - India Biodiversity Portal | **190 (29.5)**  148 (23.0)  12 (1.9)  12 (1.9)  6 (0.9)  3 (0.5)  2 (0.3)  2 (0.3)  2 (0.3)  1 (0.2)  1 (0.2)  1 (0.2) |
|  |  |
|  |  |
|  |  |
| PERSONAL COMM. and OBS.   - Personal observations - Personal communications | **172 (26.7)**  142 (22.0)  30 (4.7) |
| SCIENTIFIC PUBLICATIONS | **100 (15.5)** |
| BIODIVERSITY WEB PORTALS   - Atlas of Living Australia (ALA) - Early Detection and Distribution Mapping System (EDDMaps) - Tropicos - The National Gardening Association database (NGA) - Global Biodiversity Information Facility (GBIF) - Australian Plant Image Index (APII) - Pacific Island Ecosystems at Risk (PIER) - Texas Database - Moorea Biocode Databases - EL Verde - Flora of Gibraltar | **76 (11.8)**  41 (6.4)  17 (2.6)  4 (0.6)  3 (0.5)  2 (0.3)  2 (0.3)  2 (0.3)  2 (0.3)  1 (0.2)  1 (0.2)  1 (0.2) |
| HERBARIA and VIRTUAL HERBARIA   - New York Botanic Garden (NYBG) - Non virtual herbaria (image specimens received by email) - Atlas of Florida Plants - Intermountain - Auckland Museum* - Florida Museum of Natural History* - Alabama Plant Atlas - Biovirtual - Universidad Nacional de Colombia* - Reflora - Brazilian Plants - Chinese Virtual Herbarium (CVH) - Smithsonian Institution (US)* | **50 (7.8)**  11 (1,7)  10 (1.6)  8 (1.2)  6 (0.9)  5 (0.8)  3 (0.5)  2 (0.3)  2 (0.3)  1 (0.2)  1 (0.2)  1 (0.2) |
|  |  |
|  |  |
|  |  |
|  |  |
| PHOTOGRAPHY WEB PORTALS   - Flickr - Bisque - Dreamstime - FotoStock | **24 (3.7)**  20 (3.1)  2 (0.3)  1 (0.2)  1 (0.2) |
|  |  |
| PERSONAL BLOGS | **18 (2.8)** |
| FORUMS   - Acta Plantarum - Other forums | **11 (1.7)**  9 (1.4)  2 (0.3) |
| NEWSPAPERS | **2 (0.3)** |
| SOCIAL MEDIA   - Instagram | **1 (0.2)**  1 (0.2) |

***** Specimens virtual portals that include other organisms than plants

**Table S3.** A summary of parameters for present and future ecological niche models (ENM) performed for *Kalanchoe* × *houghtonii*. For each model is shown: the AUC scores ± standard deviation (sd), considering values higher than 0.9 as optimal results in terms of power prediction; the sensitivity, which refers to the percentage of occurrences used for modeling that are predicted inside suitable areas; the TSS (true skill statistics) ± standard deviation (sd), considering values higher than 0.5 as optimal results in terms of power prediction; the maximum sensitivity plus specificity (MSS) logistic threshold (th); and the variables in order of importance according to the jackknife test, with values of percent contribution and permutation importance or each variable within parentheses. See the text for variable details. Note that models of Future 2070 were performed with the final 20 km filtered occurrence dataset (392 records) without considering the Human Footprint (HF) variable.

| **Model** | **AUC ± sd** | **Sensitivity (%)** | **TSS ± sd** | **MSS th** | **Variables** |
| --- | --- | --- | --- | --- | --- |
| Present Raw GBIF (without HF) | 0.972±0.007 | 96.0 | 0.814±0.051 | 0.0828 | bio6 (49.7%; 70.4%) > bio5 (10.4%; 14.8%) > bio17 (22.6%; 4.8%) > bio19 (5.5%; 0.4%) > bio16 (10.3%; 3.8%) > bio8 (1.5%; 5.8%) |
| Present Raw GBIF (with HF) | 0.977±0.007 | 96.0 | 0.851±0.054 | 0.0904 | HF (35.1%; 5.4%) > bio6 (35.6%; 77.9%) > bio5 (4.7%; 7.5%) > bio17 (15.3%; 4.2%) > bio19 (4.5%; 0.6%) > bio16 (3.8%; 1.4%) > bio8 (1.0%; 2.9%) |
| Present Final (without HF) | 0.955± 0.005 | 93.9 | 0.821±0.023 | 0.1443 | bio6 (48.3%; 64.6%) > bio5 (15.2%; 16.4%) > bio19 (17.6%; 5.3%) > bio8 (4.2%; 6.7%) > bio16 (11.6%; 5.3%) > bio17 (3.1%; 1.7%) |
| Present Final (with HF) | 0.965±0.005 | 95.4 | 0.871±0.022 | 0.1101 | HF (55.9%; 22.6%) > bio6 (29.9%; 61.8%) > bio5 (7.1%; 7.6%) > bio19 (3.1%; 2.2%) > bio8 (0.9%; 2.8%) > bio16 (2.2%; 2.1%) > bio17 (1.0%; 0.9%) |
| Present Final 20 km filtered (without HF) | 0.952±0.006 | 90.8 | 0.801±0.030 | 0.1690 | bio6 (47.6%; 62.0%) > bio5 (15.5%; 17.4%) > bio19 (18.2%; 6.6%) > bio8 (4.3%; 7.7%) > bio16 (12.0%; 5.3%) > bio17 (2.3%; 1.1%) |
| Present Final 20 km filtered (with HF) | 0.967±0.005 | 94.1 | 0.848±0.025 | 0.1066 | HF (55.8%; 24.6%) > bio6 (28.0%; 56.2%) > bio5 (7.7%; 8.7%) > bio19 (3.3%; 2.6%) > bio8 (1.2%; 4.3%) > bio16 (2.7%; 2.5%) > bio17 (1.2%; 1.1%) |
| Future 2070 CCSM (RCP 2.6) | 0.953±0.007 | 88.8 | 0.804±0.030 | 0.1603 | bio6 (47.3%; 62.0%) > bio5 (14.2%; 15.6%) > bio19 (19.6%; 8.7%) > bio8 (4.5%; 6.7%) > bio16 (12.6%; 6.1%) > bio17 (1.7%; 0.8%) |
| Future 2070 CCSM (RCP 8.5) | 0.952±0.007 | 75.7 | 0.797±0.030 | 0.1690 | bio6 (46.4%; 60.0%) > bio5 (15.0%; 17.8%) > bio19 (18.5%; 7.3%) > bio8 (4.7%; 7.6%) > bio16 (12.6%; 5.4%) > bio17 (2.8%; 1.9%) |
| Future 2070 GFDL (RCP 2.6) | 0.952±0.007 | 69.1 | 0.799±0.031 | 0.1701 | bio6 (47.9%; 60.8%) > bio5 (15.1%; 15.9%) > bio19 (16.9%; 7.2%) > bio8 (4.2%; 8.0%) > bio16 (12.2%; 5.7%) > bio17 (3.7%; 2.4%) |
| Future 2070 GFDL (RCP 8.5) | 0.950±0.007 | 43.5 | 0.799±0.031 | 0.1734 | bio6 (47.0%; 57.2%) > bio5 (14.4%; 17.7%) > bio19 (18.3%; 6.6%) > bio8 (4.7%; 9.5%) > bio16 (12.7%; 6.6%) > bio17 (2.9%; 2.4%) |
| Future 2070 MPI (RCP 2.6) | 0.953±0.007 | 84.1 | 0.799±0.031 | 0.1626 | bio6 (46.4%; 58.7%) > bio5 (14.7%; 15.6%) > bio19 (18.2%; 8.4%) > bio8 (4.7%; 9.0%) > bio16 (12.8%; 6.0%) > bio17 (3.2%; 2.4%) |
| Future 2070 MPI (RCP 8.5) | 0.953±0.006 | 60.1 | 0.799±0.031 | 0.1786 | bio6 (47.0%; 60.9%) > bio5 (15.0%; 14.7%) > bio19 (18.5%; 8.3%) > bio8 (4.0%; 7.9%) > bio16 (12.8%; 6.7%) > bio17 (2.8%; 1.5%) |

**Figure S1.** Morphological variability of *Kalanchoe* × *houghtonii*. (A) and (B) adult individuals with wide and narrow leaves, respectively, from Barcelona (A) and Salou (B), Catalonia, Spain; (C) vegetative individual with deeply serrate leaves from Rangitoto Island (New Zealand); (D) the base of leaves of *K.* × *houghtonii* are sometimes inconspicuously folded (red arrows), might make identification difficult (vegetative individual from Garraf, Catalonia, Spain); (E) short rosette-vegetative individual from Pointe de la Grande Vigie, Guadeloupe (a French overseas region in the Lesser Antilles); (F) main picture, typical boat-shaped leaves of a vegetative individual in Cerro Saroche National Park (Lara State, Venezuela); inset picture, plantlets in the leaf tip, from Gavà (Catalonia, Spain) (G) main picture, inflorescence in a mature individual from Ercolano (Naples, Campania, Italy); in the inset, open flower from Floro Pérez (Holguín Province, Cuba). Photographs: (A) Carlos Gómez-Bellver; (B), (D), and inset of (F) Jordi López-Pujol; (C) Eduart Zimmer (reproduced with permission); (E) [Liliane Roubaudi](http://tela-botanica.org/profil:6865) (<https://www.tela-botanica.org/bdtxa-nn-5776-illustrations>) under license CC-BY-SA 2.0 (https://creativecommons.org/licenses/by-sa/2.0/fr/deed.en) – no changes were made by us; (F) Ileana Herrera; (G) Adriano Stinca; and inset of (G) Pedro A. González-Gutiérrez.


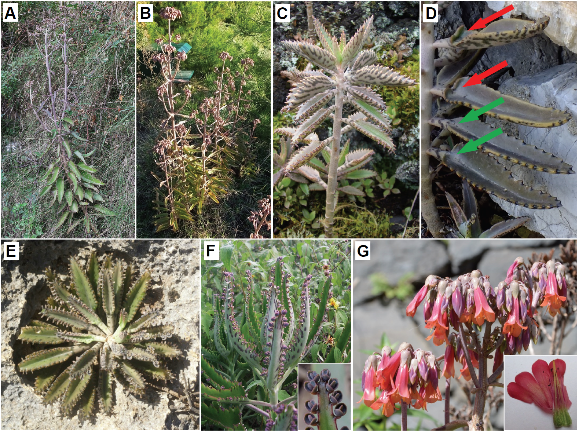


**Figure S2.** (A) illustrations of individuals of *Kalanchoe daigremontiana* (left) and *K.* × *houghtonii* (right) based on plants observed in Spain. The two species are distinguishable by the leaf base: truncate or cordate, generally conspicuously folded upwards in *K. daigremontiana*, but slightly cordate to decurrent and not folded (or doing it inconspicuously; see Supplementary Fig. S1D) in *K.* × *houghtonii* (illustrations by Carles Puche; reproduced with permission); (B) detail of the leaves of a vegetative individual of *K. daigremontiana* from Gibara (Holguín Province, Cuba; photograph: Pedro A. González-Gutiérrez); (C) vegetative individual of *K. tubiflora* from el Vendrell (Tarragona Province, Catalonia, Spain; photograph: Jordi López-Pujol); the nothospecies differs from *K. tubiflora* also by the leaves, which are narrowly linear to sub-cylindrical, sessile, with teeth, producing bulbils only at the tip.

*
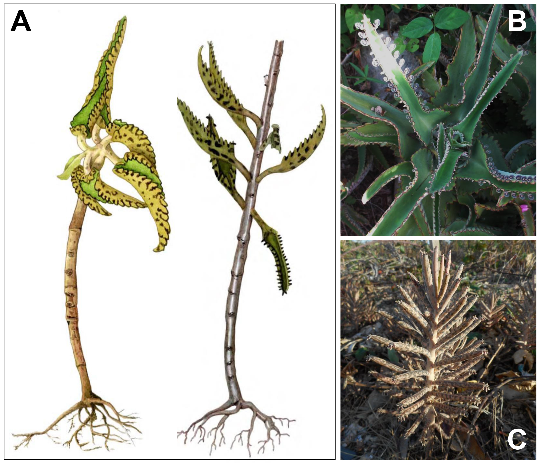
*

**Figure S3.** Variety of habitats where *Kalanchoe* × *houghtonii* can be found. (A) sandy beach (Oualie Beach, Federation of Saint Kitts and Nevis, Lesser Antilles); (B) epiphytic on a *Phoenix canariensis* trunk (Los Cancajos, La Palma, Canary Islands, Spain); (C) large infestation in Cerro Saroche National Park, in xerophytic thorny shrublands (Lara State, Venezuela); (D) growing on a path margin, in an open forest of *Pinus halepensis* (Sant Climent de Llobregat, Catalonia, Spain); (E) forming a monospecific lawn on the pavement, Sitges (Catalonia, Spain); (F) infestation on a wall in Ercolano (Naples, Campania, Italy); (G) some young individuals growing on the outer walls of Hospital Geral de Santo António (Porto, Portugal); (H) individuals growing in a building in Camagüey (Cuba); (I) young individuals growing in a fence in Caserta (Campania, Italy) (J) adult individual growing on coastal Mediterranean vegetation (Roses, Catalonia, Spain); and (K) seedlings and young individuals growing directly on the lava, Rangitoto Island (New Zealand). Photographs: (A) Susan J. Hewitt (<https://www.inaturalist.org/observations/5922976>; reproduced with permission); (B) Rainer Otto (reproduced with permission); (C) Ileana Herrera; (D) Carlos Gómez-Bellver; (E), (G), and (J) Jordi López-Pujol; (F) and (I) Adriano Stinca; (H) Pedro A. González-Gutiérrez; and (K) Eduart Zimmer (reproduced with permission).


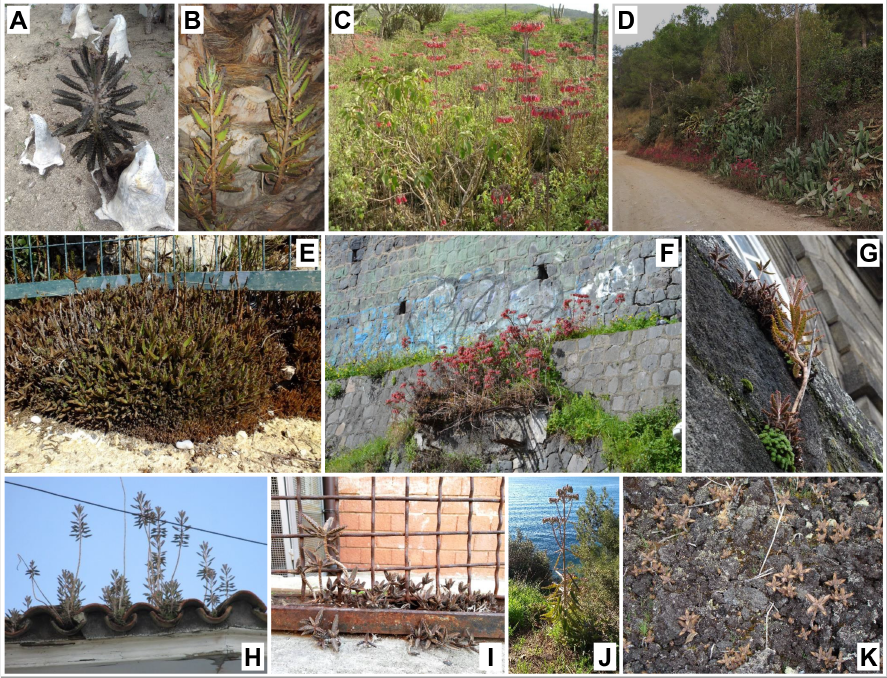


**Figure S4.** Models performed with Raw GBIF occurrence dataset (128 presence points) for *Kalanchoe* × *houghtonii* with (A) and without HF (B).


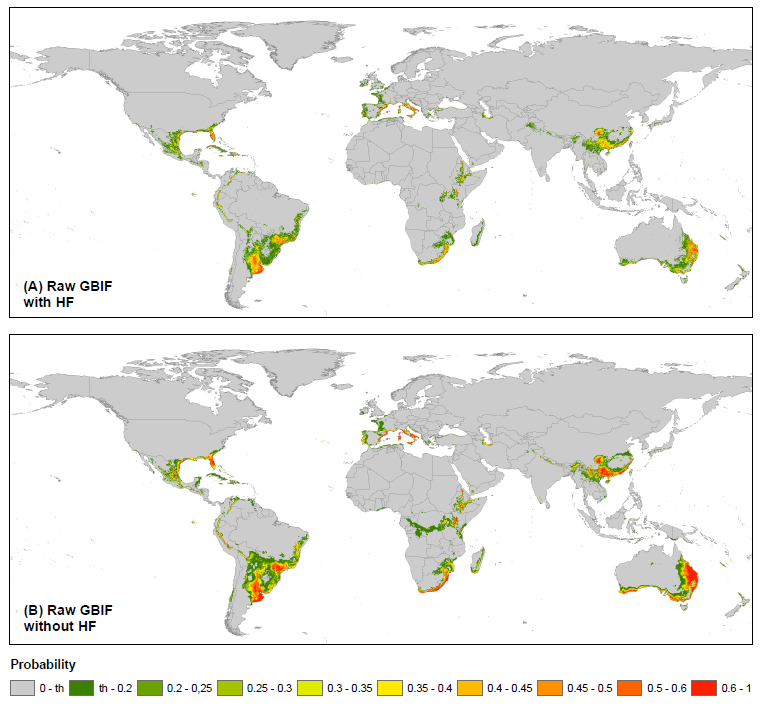


**Figure S5.** Models performed with final occurrence dataset (644 presence points) for *Kalanchoe* × *houghtonii*.


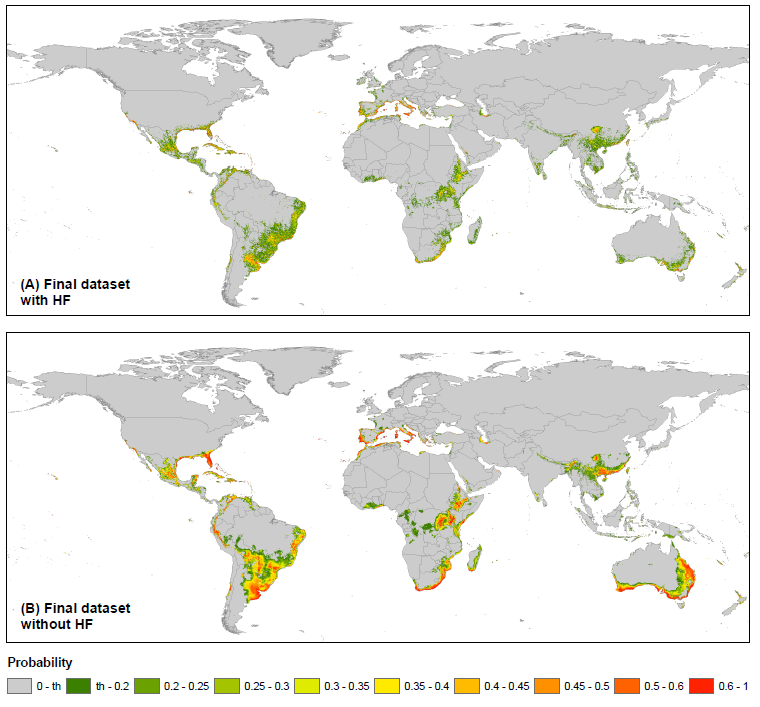


**Figure S6.** Response curves showing the effects of each variable to the present MaxEnt predictions for *Kalanchoe* × *houghtonii*. The curves show the mean response of the 100 replicate MaxEnt runs (red) and the mean +/- one standard deviation (blue, two shades for categorical variables).


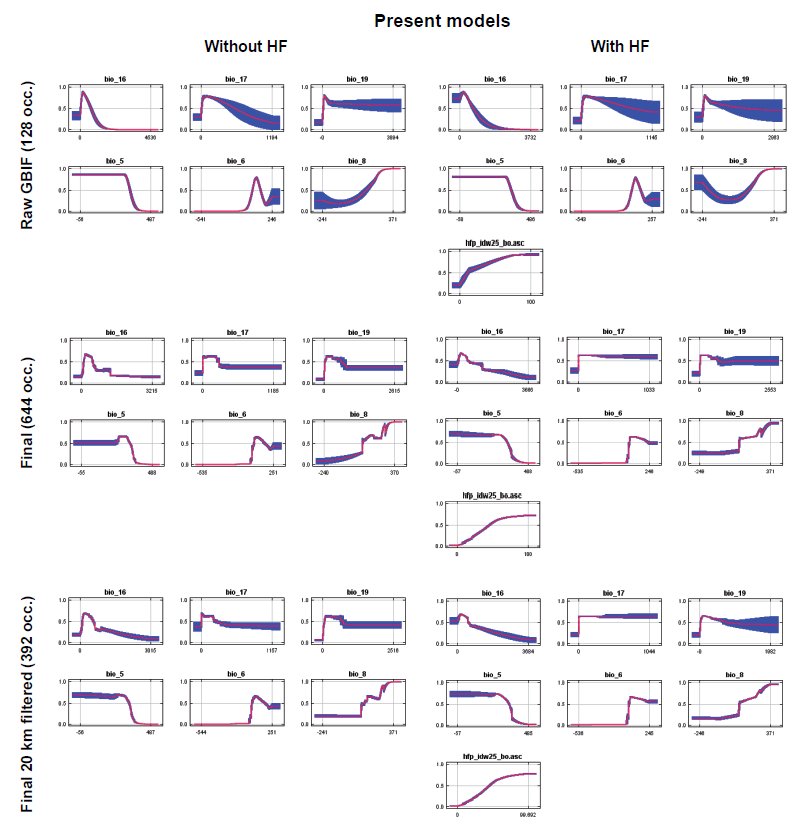


**Figure S7.** Response curves showing the effects of each variable to the future MaxEnt predictions for *Kalanchoe* × *houghtonii*. The curves show the mean response of the 100 replicate MaxEnt runs (red) and the mean +/- one standard deviation (blue, two shades for categorical variables).


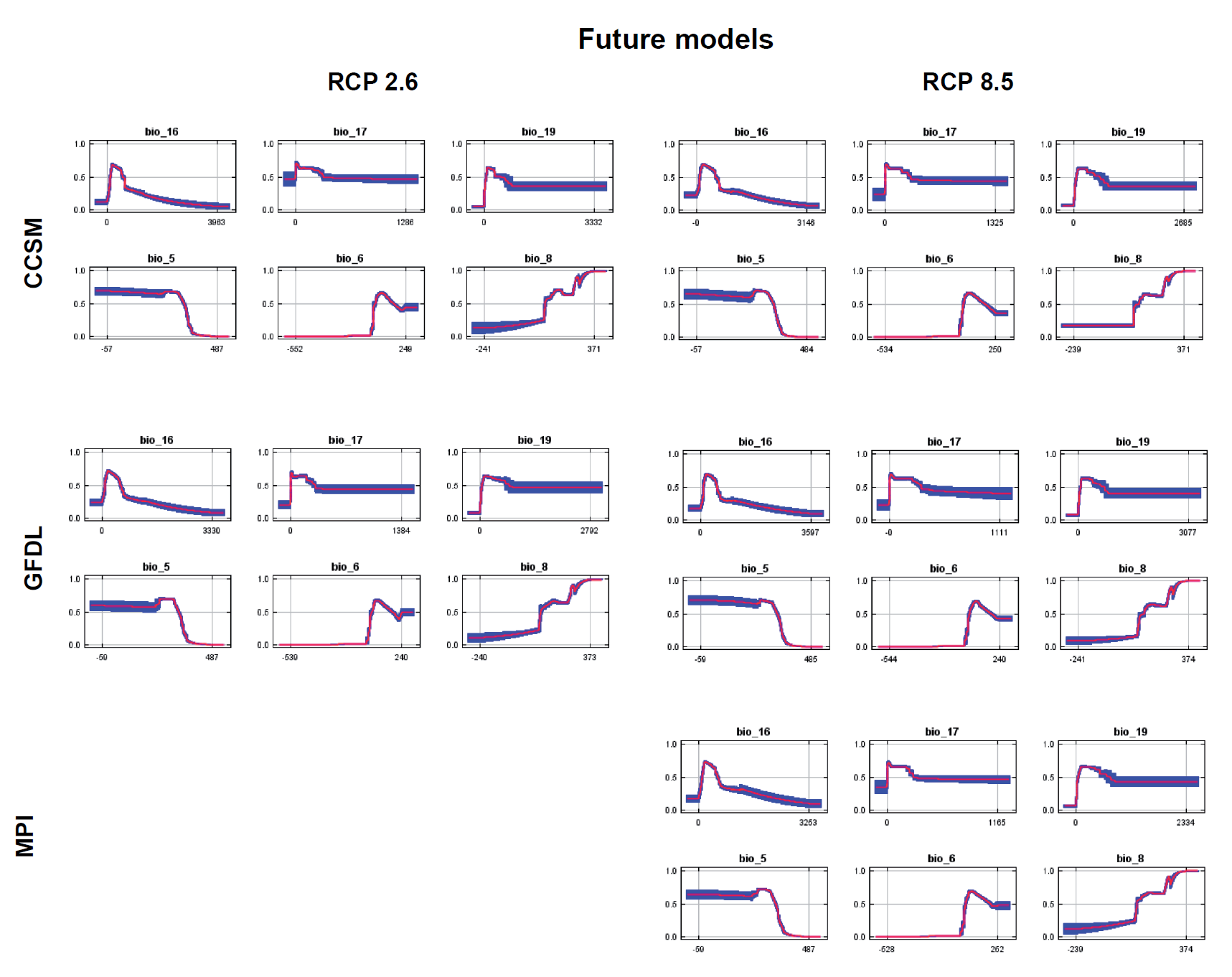


**Figure S8.** Potential distribution areas of *Kalanchoe* × *houghtonii* under future climatic conditions using the CCSM model for (A) RCP 2.6 and (B) RCP 8.5.


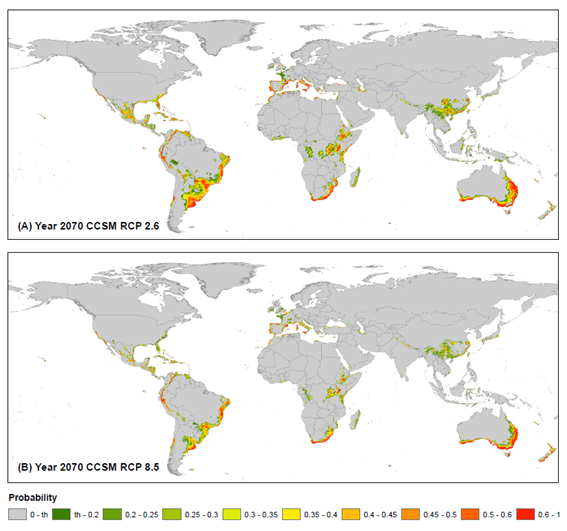


**Figure S9.** Potential distribution areas of *Kalanchoe* × *houghtonii* under future climatic conditions using the GFDL model for (A) RCP 2.6 and (B) RCP 8.5.


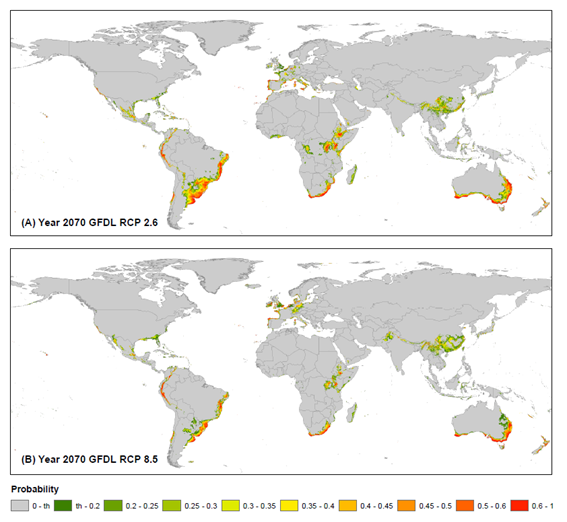


**Figure S10.** Potential distribution areas of *Kalanchoe* × *houghtonii* under future climatic conditions using the MPI model for (A) RCP 2.6 and (B) RCP 8.5.


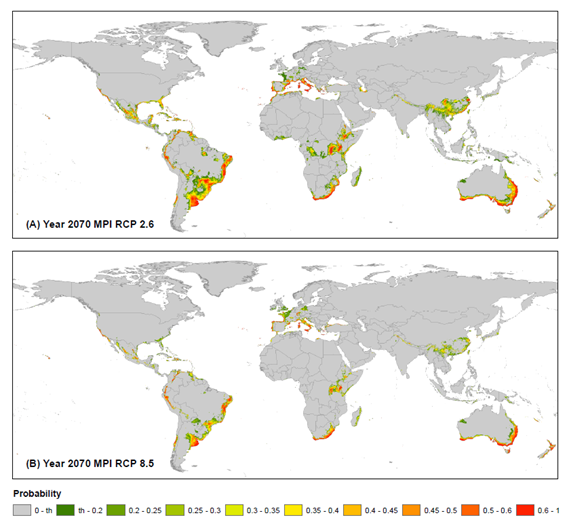


**Figure S11.** Boxplots representing values of climatic variables selected to construct the ENM models for the different suitable areas estimated: overlapped ranges between present and future model considered (in blue); lost areas for future scenarios with respect to present ranges (red); and gained areas for future scenarios with respect to present ranges (green). Note that lighter and darker colored boxplots correspond to those future models performed under scenarios 2.6 RCP and 8.5 RCP, respectively.


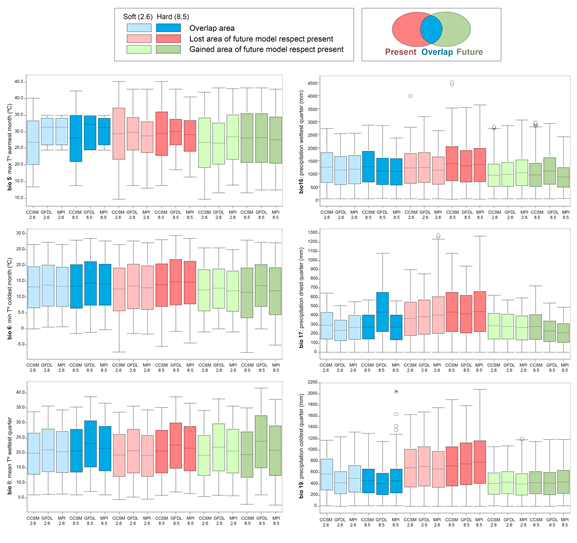

Supplement: Supplementary file 1 — Supplementary Material. [file 41598_2020_60079_MOESM1_ESM.docx]
